# Device‐Measured Physical Activity, Sedentary Behaviour and Risk of Chronic Kidney Diseases Across Levels of Grip Strength

**DOI:** 10.1002/jcsm.13726

**Published:** 2025-02-16

**Authors:** Yu He, Jing Wang, Weijie Zhang, Xinru Chen, Qiqi Wu, Yuxuan Li, Yiliang Ou, Yaping Liu, Hongliang Feng, Jihui Zhang, Sizhi Ai, Yannis Yan Liang, Yuping Ning, Jun Zhang

**Affiliations:** ^1^ Nephrology Division The Third Affiliated Hospital of Sun Yat‐sen University Guangzhou Guangdong China; ^2^ Center for Sleep and Circadian Medicine, The Affiliated Brain Hospital Guangzhou Medical University Guangzhou Guangdong China; ^3^ Key Laboratory of Neurogenetics and Channelopathies of Guangdong Province and the Ministry of Education of China Guangzhou Medical University Guangzhou Guangdong China; ^4^ The First School of Clinical Medicine Southern Medical University Guangzhou Guangdong China; ^5^ Institute of Psycho‐Neuroscience, The Affiliated Brain Hospital Guangzhou Medical University Guangzhou Guangdong China; ^6^ Geriatric Neuroscience Center, The Affiliated Brain Hospital Guangzhou Medical University Guangzhou Guangdong China; ^7^ Department of Neurology, The Affiliated Brain Hospital Guangzhou Medical University Guangzhou Guangdong China

**Keywords:** grip strength, physical activity, sedentary behaviour, triaxial accelerometer, UK Biobank

## Abstract

**Background:**

The study aimed to investigate whether the associations of accelerometer‐measured physical activity (PA) and sedentary behaviour (SB) with incident chronic kidney disease (CKD) vary based on different levels of hand grip strength, identifying the modifying role of grip strength in these associations.

**Methods:**

The study included 87 487 adults from the UK Biobank. PA and SB were quantified using accelerometers over 7‐day period, while grip strength was assessed using a hand dynamometer. CKD events were ascertained through hospital records or death registries.

**Results:**

Participants had a mean age of 62.3 years, with 57.2% (50 062) identifying as female and 97% as White. Over a median follow‐up of 7.0 years, the total incidence rate of CKD was 4.7 per 1000 person‐years. Participants who performed higher volumes of PA were more likely to be younger; have better control of body weight, blood glucose and blood pressure; and have fewer major comorbidities (*p* < 0.001). Total PA, moderate‐to‐vigorous intensity PA (MVPA), and light intensity PA (LPA), were inversely associated with CKD risk in a dose–response manner (all *p*_overall_ < 0.050). In contrast, SB was associated with a higher risk of CKD (*p*_overall_ < 0.001). Hand grip strength significantly modified the relationship between PA, SB, and CKD risk (*p*_interaction_ < 0.10). The associations of total PA (HR, 0.70; 95% CI, 0.59–0.84), MVPA (HR, 0.75; 95% CI, 0.65–0.88), LPA (HR, 0.81; 95% CI, 0.69–0.94), and SB (HR, 1.43; 95% CI, 1.21–1.69) with CKD risk were more remarkable among individuals with lower hand grip strength. Of note, physical inactivity ranked higher in relative strength for predicting CKD than hypertension, diabetes, and obesity.

**Conclusions:**

Hand grip strength could significantly modify the associations of accelerometer‐measured PA and SB with the risk of CKD. Regardless of intensity, PA consistently correlates with reduced risk of CKD, while SB is associated with increased risk, especially among individuals with lower grip strength. Notably, physical inactivity was found to be as predictive of CKD as traditional risk factors, highlighting the importance of promoting PA, especially among those with lower grip strength.

## 1 Introduction

Chronic kidney disease (CKD) has become a major global health issue, affecting over 10% of the population worldwide and ranking among the top 10 causes of death and disability [1]. With the irreversible progression of CKD, prevention through cost‐effective interventions is paramount.

Physical activity (PA) has attracted increasing attention as a promising lifestyle for the prevention of CKD [2, 3, 4]. However, studies on PA and CKD have shown mixed results, with some reported that engaging in longer leisure‐time PA was associated with a lower risk of incident CKD [5, 6, 7], whereas other studies showed little evidence [8, 9, 10]. Notably, these contradictory findings predominantly arise from studies relying on self‐reported PA, which are prone to recall and social desirability biases [11, 12]. In contrast, wearable devices such as accelerometers successfully offset recall bias of self‐reports by accurately measuring all characteristics (intensity, type, or duration) of PA, especially light intensity PA (LPA) and sedentary behaviour (SB) [13]. Previous studies indicated that the relative risk estimates derived from accelerometer‐measured PA may be almost twofold that from self‐reports [14, 15]. A recent study indicated inverse associations between both questionnaire‐based and accelerometer‐based PA and the incidence of CKD, irrespective of genetic risk [16]. However, the optimal and minimal doses and intensities of PA remain unclear.

Additionally, PA initiatives typically focus on the least active subgroup of the population [17]. To create personalized exercise programs, it is crucial to identify individuals for whom increasing PA would be especially beneficial for enhancing kidney function. PA is closely related to muscle strength, such as grip strength. Fatigue can impair physical function, making vigorous exercise difficult for some individuals [16, 18]. Notably, hand grip strength is inversely related to the incidence of CKD in the general population, and prior research has suggested that the relationship between PA and cardiovascular events is influenced by grip strength [17, 19]. However, it is unclear whether grip strength moderates the relationship between PA and CKD. In addition, SB is linked to several cardiometabolic diseases, and reducing sedentary time in favour of more active periods is a recognized target for preventing cardiovascular events [20, 21, 22]. The effects of these changes on CKD, however, remain uncertain. Additionally, insufficient PA and SB are associated with unhealthy lifestyles, mental stress, [3] or inflammatory status [23], all of which are critical for CKD prevention [3], yet the connections between these factors and the associations of PA and SB with CKD are not well understood.

To address these knowledge gaps, capitalizing on a large sample of individuals with accelerometer‐measured PA data from the UK Biobank, the first aim of the current study was to investigate the dose–response prospective associations of PA (total PA, moderate‐to‐vigorous intensity PA [MVPA], and LPA) and SB with the risk of incident CKD. Second, we aimed to clarify whether these associations were moderated by grip strength. Furthermore, we particularly compared the relative importance of physical inactivity with conventional risk factors (e.g., baseline estimated glomerular filtration rate [eGFR], coronary heart diseases, diabetes, or other lifestyles) in predicting the incidence of CKD.

## 2 Methods

### 2.1 Subjects

Subjects of the study originated from the UK Biobank study, a population‐based prospective cohort that recruited over 500 000 participants aged 37–73 years originating from 22 centres located across the United Kingdom between 2006 and 2010. All participants provided their consent for the utilization of their national health‐related hospital and death records. Both the National Health Service and the National Research Ethics Service have granted ethical approval for the UK Biobank project under reference number 11/NW/0382. More extensive details about the UK Biobank can be sourced from alternative references [24]. The flow chart was shown in Figure S1.

### 2.2 Exposures

From February 2013 to December 2015, a total of 106 053 participants agreed to wear an Axivity AX3 (Axivity, York, UK) accelerometer on their dominant wrist for seven consecutive days and 103 682 datasets were received for final data analysis. The AX3 accelerometers were initialized to collect data with a sampling frequency of 100 Hz and a dynamic range between ±8 g. The UK Biobank accelerometer expert working group calibrated the accelerometer data, while nonwear periods were identified in accordance with standard procedures [25, 26].

Additional details regarding the processing and analyses of accelerometer data have been previously outlined [27]. The total volume of PA was measured as the weekly average vector magnitude in milli‐gravity (mg) units. Additionally, PA intensity was classified using a validated accelerometer‐based activity machine learning scheme covering vigorous PA (VPA), moderate PA (MPA), and LPA [28]. Minutes per week of MPA and VPA were calculated as the sum of 5‐s epochs where the mean acceleration was ≥ 100 and ≥ 400 mg, respectively. LPA was defined as any single epoch with a mean acceleration range from 30 to 100 mg [28]. SB was defined as waking behaviour not meeting the LPA definition (mean acceleration < 30 mg) [29]. Total PA, LPA, and SB were categorized by tertiles into three groups (high, moderate and low). MVPA was divided into two groups, that is, meeting the recommended level and not meeting the recommended level, according to the standard recommendations (above 150 min per week) by the World Health Organization (WHO) [30]. The frequency of the exposures is shown in Figure S2.

Hand grip strength was measured using a Jamar J00105 hydraulic hand dynamometer while sitting. The elbow of the arm holding the dynamometer was placed against the side of the body and bent to a 90° angle with the forearm placed on an armrest. Participants were instructed to squeeze the handle of the dynamometer as hard as they could for 3 s. Both left and right hands strengths were measured. The mean of the right‐ and left‐side values, expressed as kg, was used in the analysis. The measured handgrip strength was further divided into sex‐specific tertiles: low (F: < 21.0 kg; M: < 35.5 kg), moderate (F: 21.0–26.0 kg; M: 35.5–43.0 kg) and high (F: ≥ 26.0 kg; M: ≥ 43.0 kg) [19]. Additionally, proxy indicators of sarcopenia including muscle mass and quality were also investigated [31].

### 2.3 Outcomes

The outcome of this study was incident CKD, which was defined based on hospital records or death registries (Table S1). Data were accessible up to 12 November 2021, and we implemented censorship for incident events at this specified date or at the date of the events under consideration or the occurrence of death, whichever transpired first.

### 2.4 Covariates

The following variables were potential confounding factors: age at the time of accelerometer wearing (continuous, years), sex (male/female), ethnicity (white/others), Townsend deprivation index (continuous, a score representing the deprivation of the participant's neighbourhood as a reflection of their socioeconomic position), recruitment centre (England/Wales/Scotland), educational level (degree or above/any other qualification/no qualification), season of accelerometer wear (spring/summer/autumn/winter), obesity status (underweight or normal weight/overweight/obese), healthy diet score (continuous), sleep duration (continuous, h/day), grip strength (continuous, kg), smoking status (never/previous/current), alcohol consumption (not current/two or less times a week/three or more times a week), HbA1c (continuous, mmol/mol), eGFR (continuous, mL/min per 1.73 m^2^), urate (μmol/L), C‐reactive protein (CRP), neutrophil‐to‐lymphocyte ratio (NLR), hypertension (yes/no), high cholesterol (yes/no), history of diabetes (yes/no), history of coronary heart diseases (yes/no), history of cancers (yes/no), use of blood glucose‐lowering medications (yes/no), use of blood pressure‐lowering medications (yes/no), use of cholesterol‐lowering medications (yes/no), depression (yes/no), anxiety (yes/no), use of psychotropic medications (yes/no), and ever seeking help from physicians due to anxiety or depressive symptoms (yes/no). eGFR was calculated by the Chronic Kidney Disease Epidemiology Collaboration equation (Method S1) [32]. Detailed information of covariates was described in Table S1.

### 2.5 Statistical Analysis

Statistical analyses were performed using R software Version 4.3.1 (R Development Core Team, Vienna, Austria). A value of *p* < 0.05 (two‐sided test) was considered statistically significant. Baseline characteristics, stratified by total PA categories, are displayed as *n* (%) or mean (standard deviation [SD]) or median (interquartile range [IQR]). Multiple imputations were performed to impute missing data using the ‘mice’ R package. Information about missing covariate data is shown in Table S2.

Continuous dose–response analyses assessed the shapes of associations of total/intensity‐specific PA and SB with incident CKD [33]. We used restricted cubic splines with three knots (10th, 50th and 90th) to assess the possible linear and nonlinear associations. Departure from linearity was examined by a Wald test. We defined the optimal dose as the volume of MVPA corresponding to the lowest hazard ratio (HR) (i.e., nadir of the dose curve). To provide conservative point estimates for associations, we assessed the minimal dose, defined as the volume of MVPA associated with 50% of the lowest HR [34].

Cox proportional hazards models were constructed to investigate the associations between PA and SB categories with CKD risk, with results presented as HRs and 95% confidence intervals (CIs). Additionally, the population‐attributable fraction (PAF) of CKD cases potentially preventable by eliminating physical inactivity and SB was calculated using Levin's formula (Method S1).

Interactions of PA and SB with hand grip strength were investigated using multivariable Cox regression models, including an additive or multiplicative terms. We examined the association between PA and SB with incident CKD was investigated among groups stratified by hand grip strength groups. We also investigated the interactions of PA and SB with other components of sarcopenia (muscle mass and quality), the definition and categories could be present in Method S2.

Moreover, to evaluate how important inadequate PA is in predicting CKD, the relative importance of physical inactivity (low volume of total PA) and other conventional risk factors was estimated by calculating the *R* ^2^ values of the Cox models [35]. In addition, we also evaluated the degree to which the explanatory factors accounted for the relationships between PA and SB with CKD, by calculating the percentage of excess risk mediated (PERM) for several categories of explanatory variables. Detailed information was shown in Method S3.

In subgroup analyses, we repeated the main analyses stratified by age (< 65/≥ 65 years), sex (female/male), obesity status (underweight or normal weight/overweight/obese) and eGFR levels (≥ 90/< 90 mL/min per 1.73 m^2^). To ensure the robustness of our findings, we conducted several sensitivity analyses. These included the use of Fine–Gray subdistribution hazard models to account for death as a competing risk for incident CKD and additional analyses excluding participants with any missing covariate data and events occurring within the first 2 years of follow‐up.

## 3 Results

### 3.1 Baseline Characteristics

The study included 87 487 participants (57.0% female, mean age 62.3 years, 97.0% White ethnicity). As summarized in Tables 1 and S3–S5, the participants' characteristics are displayed across the levels of PA and SB. Of the study population, 33.3% (29 176) of participants engaged in total PA in a low volume, 33.4% (29 184) in a moderate volume and 33.3% (29 127) in a high volume; 39.7% (34 748) met the recommended level for MVPA. Participants with moderate and high volumes of total PA were more likely to be younger and better educated; less current use of tobacco or alcohol; have better control of body weight, blood glucose and blood pressure; have less likelihood of depression and anxiety; and have fewer major comorbidities than those with low volumes of total PA (*p* < 0.05). Similar patterns are seen among individuals meeting or not meeting the recommended level for MVPA.

**TABLE 1:** Baseline characteristics of the study participants stratified by levels of total physical activity (*N* = 87 487).

Characteristics	Overall (*N* = 87 487)	Total PA (mg)			*p*
		Low (*N* = 29 176)	Moderate (*N* = 29 184)	High (*N* = 29 127)	
Age at accelerometry (years), mean (SD)	62.3 (7.8)	64.6 (7.4)	62.3 (7.7)	59.9 (7.7)	< 0.001
Female, *n* (%)	50 062 (57.2)	15 381 (52.7)	17 214 (59.0)	17 467 (60.0)	< 0.001
White ethnicity, *n* (%)	84 855 (97.0)	28 422 (97.5)	28 336 (97.1)	28 077 (96.4)	< 0.001
Townsend deprivation index, median [IQR]^a^	−2.5 [−3.8, −0.2]	−2.4 [−3.8, −0.0]	−2.5 [−3.9, −0.3]	−2.5 [−3.8, −0.3]	< 0.001
Recruitment centre, *n* (%)					0.082
England	78 502 (89.7)	26 200 (89.8)	26 210 (89.8)	26 092 (89.6)	
Scotland	3293 (3.8)	1145 (3.9)	1080 (3.7)	1068 (3.7)	
Wales	5692 (6.5)	1813 (6.3)	1894 (6.5)	1967 (6.8)	
Education level, *n* (%)					< 0.001
Degree or above	38 497 (44.0)	12 250 (42.0)	13 068 (44.8)	13 179 (45.2)	
Any other qualification	41 831 (47.8)	13 945 (47.8)	13 875 (47.5)	14 011 (48.1)	
No qualification	7159 (8.2)	2981 (10.2)	2241 (7.7)	1937 (6.7)	
Season of accelerometer wear, *n* (%)					< 0.001
Spring	19 764 (22.6)	6148 (21.1)	6576 (22.5)	7040 (24.2)	
Summer	22 825 (26.1)	6998 (24.0)	7626 (26.1)	8201 (28.2)	
Autumn	26 171 (29.9)	8902 (30.5)	8754 (30.0)	8515 (29.2)	
Winter	18 727 (21.4)	7128 (24.4)	6228 (21.3)	5371 (18.4)	
Obesity status, *n* (%)					< 0.001
Underweight or normal weight	35 151 (40.2)	8294 (28.4)	11 620 (39.8)	15 237 (52.3)	
Overweight	35 938 (41.1)	12 608 (43.2)	12 523 (42.9)	10 807 (37.1)	
Obese	16 398 (18.7)	8274 (28.4)	5041 (17.3)	3083 (10.6)	
Healthy diet score, median [IQR]	3.0 [2.0, 4.0]	3.0 [2.0, 3.0]	3.0 [2.0, 4.0]	3.0 [2.0, 4.0]	< 0.001
Sleep duration (h/day), mean (SD)	7.3 (0.9)	7.5 (0.9)	7.3 (0.9)	7.1 (0.9)	< 0.001
Grip strength (kg), mean (SD)	30.3 (10.6)	30.2 (10.9)	30.0 (10.6)	30.6 (10.3)	< 0.001
Smoking status, *n* (%)					< 0.001
Never	50 565 (57.8)	15 995 (54.8)	17 069 (58.5)	17 501 (60.1)	
Previous	31 463 (36.0)	10 961 (37.6)	10 434 (35.8)	10 068 (34.5)	
Current	5459 (6.2)	2220 (7.6)	1681 (5.8)	1558 (5.3)	
Alcohol consumption, *n* (%)					< 0.001
Not current	5158 (5.9)	1981 (6.8)	1578 (5.4)	1599 (5.5)	
Two or less times a week	40 414 (46.2)	14 083 (48.3)	13 257 (45.4)	13 074 (44.9)	
Three or more times a week	41 915 (47.9)	13 112 (44.9)	14 349 (49.2)	14 454 (49.6)	
HbA1c (mmol/mol), mean (SD)	35.3 (5.3)	36.1 (6.3)	35.1 (4.9)	34.7 (4.4)	< 0.001
HbA1c (%), mean (SD)	5.4 (0.5)	5.5 (0.6)	5.4 (0.5)	5.3 (0.4)	< 0.001
eGFR (mL/min per 1.73 m^2^), mean (SD)	92.0 (7.4)	90.1 (7.3)	92.0 (7.3)	93.8 (7.3)	< 0.001
Urate (μmol/L), mean (SD)	301.0 (77.1)	315.3 (78.8)	299.5 (75.9)	288.2 (74.0)	< 0.001
CRP (mg/L), mean (SD)	2.20 (3.8)	2.7 (4.3)	2.1 (3.6)	1.7 (3.3)	< 0.001
NLR (%), mean (SD)	2.33 (1.2)	2.4 (1.2)	2.3 (1.1)	2.3 (1.2)	< 0.001
Hypertension, *n* (%)	22 576 (25.8)	10 246 (35.1)	7229 (24.8)	5101 (17.5)	< 0.001
High cholesterol, *n* (%)	11 822 (13.5)	5586 (19.1)	3839 (13.2)	2397 (8.2)	< 0.001
Depression, *n* (%)	7714 (8.8)	2842 (9.7)	2522 (8.6)	2350 (8.1)	< 0.001
Anxiety, *n* (%)	767 (0.9)	348 (1.2)	238 (0.8)	181 (0.6)	< 0.001
Seeking help from physicians, *n* (%)	33 675 (38.5)	11 402 (39.1)	11 200 (38.4)	11 073 (38.0)	0.027
History of diabetes, *n* (%)	3763 (4.3)	2153 (7.4)	990 (3.4)	620 (2.1)	< 0.001
History of coronary heart diseases, *n* (%)	4075 (4.7)	2164 (7.4)	1226 (4.2)	685 (2.4)	< 0.001
History of cancers, *n* (%)	12 451 (14.2)	5001 (17.1)	4148 (14.2)	3302 (11.3)	< 0.001
Medication usage, *n* (%)					
Use of blood glucose‐lowering medications	2056 (2.4)	1248 (4.3)	507 (1.7)	301 (1.0)	< 0.001
Use of cholesterol‐lowering medications	14 358 (16.4)	6956 (23.8)	4599 (15.8)	2803 (9.6)	< 0.001
Use of blood pressure‐lowering medications	15 906 (18.2)	7647 (26.2)	5044 (17.3)	3215 (11.0)	< 0.001
Use of psychotropic medications	2024 (2.3)	900 (3.1)	633 (2.2)	491 (1.7)	< 0.001

*Note:* Data are presented as mean (SD), median [IQR] or *n* (%).

Abbreviations: CRP, C‐reactive protein; eGFR, estimated glomerular filtration rate; IQR, interquartile range; NLR, neutrophil‐to‐lymphocyte ratio; SD, standard deviation.

^a^ Townsend deprivation index was calculated based on the preceding national census output areas prior to participants joining UK Biobank.

### 3.2 Associations of PA and SB With Incident CKD

**FIGURE 1:**
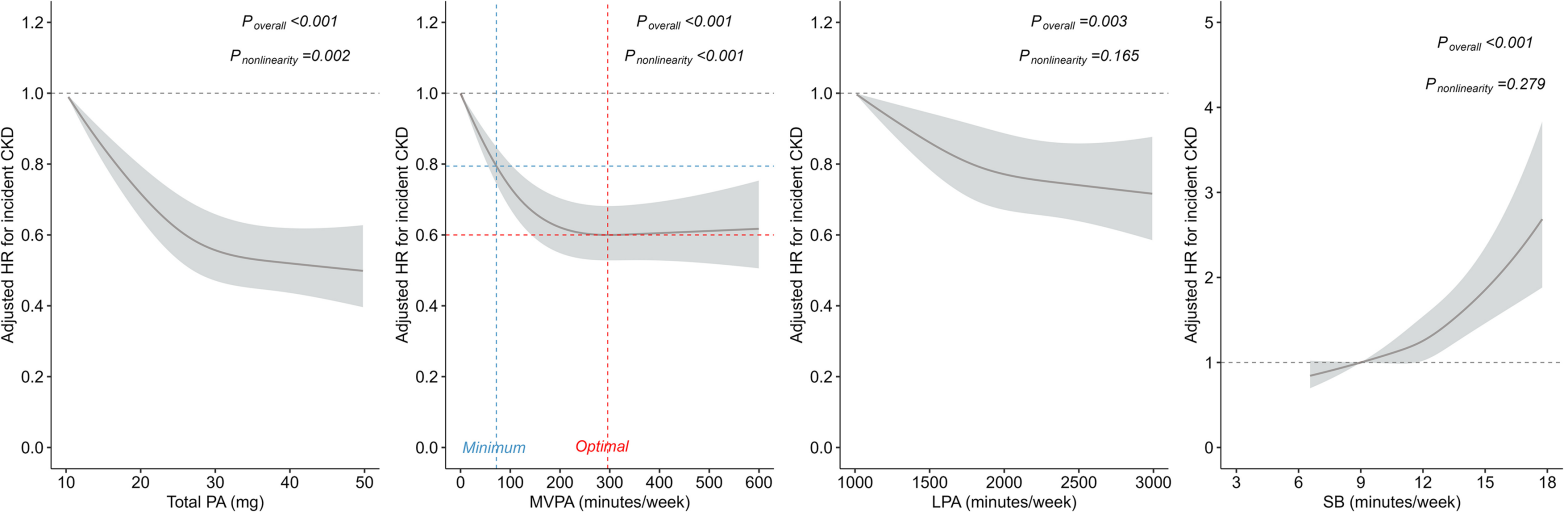
Dose–response associations of the accelerometer‐measured total volume of PA, MVPA, LPA and SB with incident CKD. The solid line referred to the HRs from restricted cubic spline regression. Restricted cubic splines were constructed with three knots. Adjusted HRs (95% CI) were calculated using Cox proportional hazards regression analysis adjusted for age at the time of accelerometer wearing, sex, ethnicity, Townsend deprivation index, recruitment centre, education level, the season of accelerometer wearing, obesity status, healthy diet score, sleep duration, grip strength, smoking status, alcohol consumption, HbA1c, eGFR, urate, CRP, NLR, hypertension, high cholesterol, history of diabetes, history of coronary heart diseases, history of cancers, use of blood glucose‐lowering medications, use of blood pressure‐lowering medications, use of cholesterol‐lowering medications, depression, anxiety, use of psychotropic medications and ever seeking help from physicians due to anxiety or depressive symptoms. CKD, chronic kidney disease; CI, confidence interval; CRP, C‐reactive protein; eGFR, estimated glomerular filtration rate; HR, hazard ratio; LPA, light intensity PA; MVPA, moderate‐to‐vigorous intensity PA; NLR, neutrophil‐to‐lymphocyte ratio; PA, physical activity; SB, sedentary behaviour.

Over a median follow‐up of 7.0 years (IQR, 6.4–7.5), 2820 CKD cases (overall incidence rate: 4.7 per 1000 person‐years) were documented. As indicated in Figure 1, the associations of total PA and MVPA with incident CKD were nonlinear (*p*_nonlinearity_ < 0.05), whereas undertaking LPA had no maximal or minimal dose for reducing CKD risk (*p*_nonlinearity_ = 0.165). A substantial reduction in CKD risk was observed at a minimal dose of 72.1 min/week of MVPA (HR, 0.79; 95% CI, 0.74–0.85), while the lowest CKD risk (HR, 0.59; 95% CI, 0.53–0.68) was achieved at 295.9 min/week of MVPA and then plateaued thereafter.

In Table 2, for total PA, compared with the group with a low volume, those with a high volume demonstrated a 26% (HR, 0.74; 95% CI, 0.67–0.83) reduced risk of incident CKD; meeting the recommended level for MVPA was associated with a 18% (HR, 0.82; 95% CI, 0.75–0.90) lower risk of developing CKD relative to not meeting the recommended level for MVPA; for LPA in a high volume compared with a low volume was significantly associated with a reduced risk of developing CKD (HR, 0.89; 95% CI, 0.80–0.98). SB was associated with higher risk of incident CKD (HR, 1.26; 95% CI, 1.13–1.40).

**TABLE 2:** Associations of the accelerometer‐measured total volume of PA, MVPA, LPA and SB with incident CKD.

Exposures	*N*	Incident rate (per 1000 person‐years)	Model 1^a^	Model 2^b^	Model 3^c^	PAF (%)
			HR (95% CI)	HR (95% CI)	HR (95% CI)	
Total PA						10.4
Low	29 176	7.2	1 (reference)	1 (reference)	1 (reference)	
Moderate	29 184	4.3	0.71 (0.65–0.77)	0.83 (0.76–0.91)	0.88 (0.80–0.96)	
High	29 127	2.7	0.53 (0.47–0.58)	0.68 (0.61–0.76)	0.74 (0.67–0.83)	
MVPA						11.7
Not meeting the recommended level	52 739	5.8	1 (reference)	1 (reference)	1 (reference)	
Meeting the recommended level	34 748	3.0	0.63 (0.57–0.68)	0.78 (0.71–0.85)	0.82 (0.75–0.90)	
LPA						4.2
Low	29 162	5.7	1 (reference)	1 (reference)	1 (reference)	
Moderate	29 163	4.7	0.87 (0.80–0.95)	0.96 (0.88–1.05)	1.00 (0.91–1.09)	
High	29 162	3.8	0.73 (0.67–0.80)	0.85 (0.77–0.94)	0.89 (0.80–0.98)	
SB						7.8
Low	29 163	3.7	1 (reference)	1 (reference)	1 (reference)	
Moderate	29 162	4.4	1.15 (1.05–1.27)	1.09 (0.99–1.21)	1.07 (0.97–1.18)	
High	29 162	6.1	1.52 (1.38–1.67)	1.33 (1.19–1.47)	1.26 (1.13–1.40)	

Abbreviations: CKD, chronic kidney disease; CI, confidence interval; CRP, C‐reactive protein; eGFR, estimated glomerular filtration rate; HR, hazard ratio; LPA, light intensity PA; MVPA, moderate‐to‐vigorous intensity PA; NLR, neutrophil‐to‐lymphocyte ratio; PA, physical activity; PAF, population‐attributable fraction; SB, sedentary behaviour.

^a^ Model 1 was adjusted for age at the time of accelerometer wearing and sex.

^b^ Model 2 was adjusted for age at the time of accelerometer wearing, sex, ethnicity, Townsend deprivation index, recruitment centre, education level, the season of accelerometer wearing, obesity status, healthy diet score, sleep duration, grip strength, smoking status, alcohol consumption, HbA1c, eGFR, urate, CRP and NLR.

^c^ Model 3 was adjusted for age at the time of accelerometer wearing, sex, ethnicity, Townsend deprivation index, recruitment centre, education level, the season of accelerometer wearing, obesity status, healthy diet score, sleep duration, grip strength, smoking status, alcohol consumption, HbA1c, eGFR, urate, CRP, NLR, hypertension, high cholesterol, history of diabetes, history of coronary heart diseases, history of cancers, use of blood glucose‐lowering medications, use of blood pressure‐lowering medications, use of cholesterol‐lowering medications, depression, anxiety, use of psychotropic medications and ever seeking help from physicians due to anxiety or depressive symptoms.

Table 2 also shows the proportions of CKD events that could have been prevented by undertaking PA. Assuming the associations to be causal, which cannot be concluded in the present study, 10.4% of new‐onset CKD events in this study population were attributed to maintaining a low volume of total PA, 11.7% to maintaining MVPA under the recommended level, 7.8% to maintaining SB and 4.2% to maintaining a low volume of LPA.

### 3.3 Interaction of PA and SB With Hand Grip Strength for CKD

There was a significant interaction between grip strength and PA (total PA, *p*_multiplicative interaction_ = 0.311, *p*_additive interaction_ = 0.013; MVPA, *p*_multiplicative interaction_ = 0.420, *p*_additive interaction_ = 0.025; LPA, *p*_multiplicative interaction_ = 0.139, *p*_additive interaction_ = 0.082). The association between grip strength and SB showed similar pattern (*p*_multiplicative interaction_ = 0.097, *p*_additive interaction_ = 0.005). When stratified by levels of grip strength, the reduction in risk of CKD associated with total PA was more pronounced among participants with low grip strength (high vs. low: HR, 0.70; 95% CI, 0.59–0.84) or moderate (high vs. low: HR, 0.73; 95% CI, 0.61–0.88) grip strength in contrast with high (high vs. low: HR, 0.82; 95% CI, 0.66–1.01) (Table 3).

**TABLE 3:** Associations of the accelerometer‐measured total volume of PA, MVPA, LPA and SB with incident CKD stratified by different levels of grip strength.

Exposures	Low grip strength			Moderate grip strength			High grip strength		
	*N*	Incident rate (per 1000 person‐years)	HR (95% CI)	*N*	Incident rate (per 1000 person‐years)	HR (95% CI)	*N*	Incident rate (per 1000 person‐years)	HR (95% CI)
Total PA									
Low	11 520	8.1	1 (reference)	9608	7.7	1 (reference)	8048	5.3	1 (reference)
Moderate	10 235	5.1	0.87 (0.76–1.00)	9818	4.4	0.86 (0.74–1.00)	9131	3.4	0.91 (0.76–1.10)
High	8862	3.1	0.70 (0.59–0.84)	9883	2.7	0.73 (0.61–0.88)	10 382	2.3	0.82 (0.66–1.01)
*p*_multiplicative interaction_									0.311
*p*_additive interaction_									0.013
MVPA									
Not meeting the recommended level	20 004	6.8	1 (reference)	17 354	6.0	1 (reference)	15 381	4.4	1 (reference)
Meeting the recommended level	10 613	3.4	0.75 (0.65–0.88)	11 955	3.3	0.86 (0.74–1.00)	12 180	2.5	0.86 (0.72–1.02)
*p*_multiplicative interaction_									0.420
*p*_additive interaction_									0.025
LPA									
Low	10 099	7.0	1 (reference)	9831	5.8	1 (reference)	9232	4.2	1 (reference)
Moderate	10 023	5.7	0.98 (0.86–1.13)	9915	4.8	1.04 (0.89–1.21)	9225	3.5	0.97 (0.80–1.16)
High	10 495	4.3	0.81 (0.69–0.94)	9563	4.1	0.99 (0.84–1.17)	9104	2.9	0.90 (0.73–1.10)
*p*_multiplicative interaction_									0.139
*p*_additive interaction_									0.082
SB									
Low	9970	4.2	1 (reference)	9635	3.8	1 (reference)	9558	3.0	1 (reference)
Moderate	10 128	5.1	1.08 (0.92–1.27)	9792	4.7	1.09 (0.92–1.29)	9242	3.3	1.03 (0.84–1.25)
High	10 519	7.5	1.43 (1.21–1.69)	9882	6.1	1.12 (0.93–1.34)	8761	4.3	1.19 (0.96–1.47)
*p*_multiplicative interaction_									0.097
*p*_additive interaction_									0.005

*Note:* Hazard ratio was adjusted age for at the time of accelerometer wearing, sex, ethnicity, Townsend deprivation index, recruitment centre, education level, the season of accelerometer wearing, obesity status, healthy diet score, sleep duration, grip strength, smoking status, alcohol consumption, HbA1c, eGFR, urate, CRP, NLR, hypertension, high cholesterol, history of diabetes, history of coronary heart diseases, history of cancers, use of blood glucose‐lowering medications, use of blood pressure‐lowering medications, use of cholesterol‐lowering medications, depression, anxiety, use of psychotropic medications and ever seeking help from physicians due to anxiety or depressive symptoms.

Abbreviations: CKD, chronic kidney disease; CI, confidence interval; CRP, C‐reactive protein; eGFR, estimated glomerular filtration rate; HR, hazard ratio; LPA, light intensity PA; MVPA, moderate‐to‐vigorous intensity PA; NLR, neutrophil‐to‐lymphocyte ratio; PA, physical activity; SB, sedentary behaviour.

As Table 3 shows, the associations of MVPA and LPA with the risk of CKD also varied across different hand grip strength levels, with maximum reduction of CKD risk at the low grip strength level for MVPA meeting the recommended level and high LPA. Moreover, SB could increase more risk of incident CKD among individuals with low grip strength (high vs. low: HR, 1.43; 95% CI, 1.21–1.69) than those with moderate (high vs. low: HR, 1.12; 95% CI, 0.93–1.34) and high grip strength (high vs. low: HR, 1.19; 95% CI, 0.96–1.47). The interactions between muscle mass, quality, PA and SB are shown in Tables S6–S8. Muscle mass and quality may potentially modify the associations of PA and SB with the risk of CKD even though the interactions were not statistically significant.

### 3.4 Relative Importance of Physical Inactivity Compared With Other Traditional Risk Factors in Predicting CKD

**FIGURE 2:**
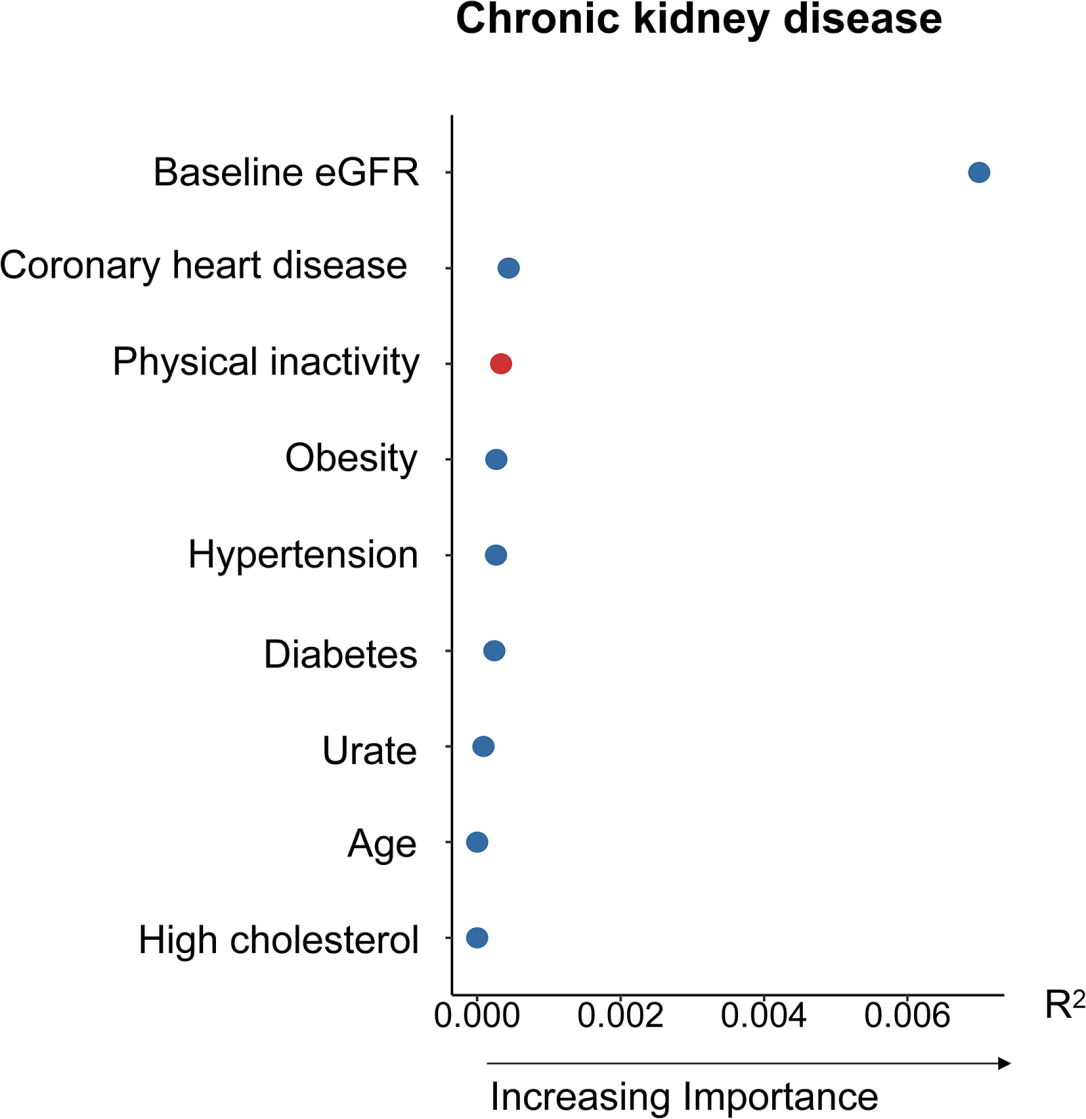
The relative importance of total volume of PA and other traditional risk factors in predicting CKD risk. CKD, chronic kidney disease; eGFR, estimated glomerular filtration rate; PA, physical activity.

Figure 2 displays the relative importance of physical inactivity (low volume of total PA) in predicting CKD compared with other selected conventional risk factors. As indicated by *R* ^2^, the baseline eGFR ranked the first among this list of selected risk factors for CKD risk, while physical inactivity ranked third, just slightly lower than the history of coronary heart diseases, but higher than obesity, hypertension, diabetes, urate, age, and high cholesterol.

### 3.5 Explanatory Factors, Subgroup and Sensitivity Analyses

As shown in Figure S3, the HR for total PA was 0.53 (95% CI, 0.48–0.59) after minimal adjustment (i.e., adjustment for age, sex, ethnicity, assessment centre, and socioeconomic factors) and decreased by nearly 45% after adjustment all risk factors. Physiological factors (PERM 28%), comorbidities and medication (PERM 19%) were the primary explanatory factors for the association between total PA and an increased risk of developing CKD. Additionally, the association was also explained by health behaviours (PERM 2%), inflammatory factors (PERM 2%), and psychological factors (PERM 2%). In terms of MVPA, LPA, and SB, the patterns were similar with the total PA group.

The associations between levels of total PA and incident CKD risk were generally consistent across subgroups stratified by age (< 65 and ≥ 65 years), sex (female and male), obesity status (underweight or normal and overweight), and eGFR level (≥ 90 and < 90 mL/min per 1.73 m^2^) (Table S9). In the sensitivity analyses, the associations of total PA, MVPA, LPA, and SB with new‐onset CKD remained rather robust when considering competing risk of death (Table S10), the associations of total PA and MVPA with incident CKD remained stable after using the sample with complete cases (Figure S4 and Table S11), and excluding the CKD events occurring within the first 2 years (Figure S5 and Table S12), respectively.

## 4 Discussion

To the best of our knowledge, based on the largest accelerometry dataset to date, the present study has several noteworthy findings. First, accelerometer‐measured PA, especially MVPA, was associated with a lower risk of CKD, whereas SB was associated with an elevated risk of CKD. Most importantly, our study provided the first evidence that hand grip strength could modify the associations of PA and SB with the risk of CKD. Notably, the associations of PA and SB with incident CKD were more remarkable among individuals with low hand grip strength. Furthermore, we demonstrated that physical inactivity is among the top three traditional risk factors for CKD, along with factors such as reduced eGFR, coronary heart disease, obesity, and hypertension [3].

In line with the most previous studies [5, 36, 37, 38] and the current PA guidelines [30], our study emphasizes that MVPA is significantly more efficient than LPA in reducing CKD risk. We determined that the PAF for MVPA was nearly double that of LPA, highlighting its superior benefit in lowering CKD risk. A previous study reported an equivalent contribution of accelerometer‐measured guideline‐recommended moderate PA to heart failure [36], while a lower PAF was noted for self‐reported total PA in individuals with type 2 diabetes [2]. Nevertheless, as ‘every movement counts’, LPA remains a viable option for individuals who may find vigorous exercise too challenging due to fatigue or physical limitations [39]. This approach is particularly beneficial for those at high risk of developing CKD, as evidenced by a cohort study which found that increased LPA, assessed via self‐reports, was linked with a reduced long‐term risk of CKD [5].

Notably, we delineated curvilinear dose–response patterns for total PA and MVPA, with minimal and optimal doses, owing to the advantage of collecting point‐to‐point PA data from accelerometers. The threshold of MVPA (optimal dose: 295.9 min/week) assessed by the accelerometer in our study suggested that taking MVPA beyond the current guideline‐recommended level (150 min/week) would derive extra benefits. While for those unable to achieve this amount, it is encouraging to note that just meeting a minimal dose of MVPA (at least 72 min/week), far lower than guideline‐recommended level, could have gained approximately 20% lower risk for incident CKD. A recent study indicated that engaging in multiple types of accelerometer‐measured PA were associated with a lower risk of developing CKD regardless of genetic risk [16]. However, the previous study overlooked the detrimental effects of SB and did not account for the role of muscle strength in moderating PA's impact on CKD risk reduction.

Individuals who often feel of fatigue usually experience impaired physical function, making it challenging for them to tolerate more vigorous exercise [19]. Hand grip strength, a reliable measure of limb muscle strength across all ages, has been shown to be significantly associated with activity levels [40] and incident CKD [19]. This suggests that grip strength not only reflects physical function but may also influence the relationship between PA and health outcomes. A previous study reported that the association between PA and risk of mortality was modulated by grip strength [17]. Our findings underscore the significant interplay, particularly among individuals with lower grip strength. This interaction highlights the potential of tailored PA programmes that consider individual strength capacities to effectively mitigate CKD risk.

The beneficial impact of PA on CKD might occur through several mechanisms. First, PA may reduce adipocytokines, leading to decreased angiotensinogen levels and improved kidney endothelium function [S1]. Additionally, enhancements of renal vasculature and insulin sensitivity could also explain the positive effects of PA on kidney function [6]. The putative relationship between the PA and risk of CKD might also be mediated through indirect pathways. Specifically, physical inactivity could increase the risk of other traditional risk factors of CKD. For instance, individuals who are physically inactive are more likely to be obese and may have poor control over blood pressure [S2], blood glucose [S3], uric acid levels [S4] and cholesterol [S5]. Consequently, physical inactivity could heighten the risk of CKD through both direct and indirect pathways, potentially exerting a greater influence than other individual traditional risk factors.

### 4.1 Clinical Implications

This study enhances our understanding by positioning PA among the top three risk factors for CKD, surpassing other established risk factors such as hypertension, diabetes and obesity. Supporting this, a prior analysis from the ONTARGET study revealed that enhancing PA exerted the most significant impact on preventing CKD onset and reducing mortality compared to other lifestyle factors among adults with type 2 diabetes [2]. Coupled with prior evidence suggesting causality [4], we advocate that maintaining sufficient PA, particularly MVPA, is a cost‐effective approach for mitigating CKD burden. For those unable to engage in more vigorous activities, opting for LPA, such as slow walking or light housework, may present a feasible alternative that could help delay CKD onset. Our findings also highlight that encouraging more PA and reducing SB among individuals with low hand grip strength may significantly enhance the prevention of CKD. Hand grip strength is easy to measure and highly reproducible, making it an effective screening tool in routine clinical practice. This can help identify individuals for whom increasing PA would be particularly beneficial for improving kidney function.

### 4.2 Limitations

Although based on a large longitudinal dataset with objectively measured PA and SB, the study findings should be interpreted in mind of several limitations. First, the present analyses of the UK Biobank study only included White participants; therefore, the current findings are expected to be corroborated by other ethnicities. Second, the present study failed to capture time‐varying PA, despite PA habits are generally considered relatively stable over time [S6]. Third, the follow‐up period was relatively short as accelerometry data were collected between 2013 and 2015. To address reverse causation, we excluded events occurring within the first 2 years and obtained robust findings. Last, because of the observational study design, the presence of unmeasured confounding factors cannot be ruled out despite a comprehensive consideration of covariates.

## 5 Conclusion

The study underscores the importance of maintaining sufficient PA and reducing SB to mitigate CKD risk, particularly among individuals with lower baseline grip strength. Notably, insufficient PA may pose a comparable CKD risk to other traditional factors. Future guidelines and policies should prioritize promoting PA and discouraging SB for CKD prevention, especially among those with lower grip strength levels.

## Conflicts of Interest

J.Z. is funded by the National Natural Science Foundation Project Cultivation Special Fund of the Third Affiliated Hospital of Sun Yat‐sen University (2022GZRPYMS09). J.W. is supported by Guangzhou Science and Technology Program (2023A03J0829), and Guangdong Basic and Applied Basic Research Foundation (2024A1515011349). Other co‐authors declared that there are no conflicts of interest.

## Supporting information


**Figure S1.** Flow chart of enrolment.
**Method S1.** Formulas.
**Method S2.** Detailed information of the muscle mass and quality.
**Method S3.** Detailed information of the percentage of excess risk mediated (PERM).
**Figure S2.** Frequency of total PA, MVPA, LPA and sedentary behaviour (SB).
**Table S1.** Information about exposures, outcomes, covariates and mediators.
**Table S2.** The number (percentage) of participants with missing covariate data in the analytic sample.
**Table S3.** Baseline characteristics of the study participants stratified by MVPA.
**Table S4.** Baseline characteristics of the study participants stratified by LPA.
**Table S5.** Baseline characteristics of the study participants stratified by SB.
**Figure S3.** Proportions attributable to different risk factors of CKD.
**Table S6.** Associations of the accelerometer‐measured total volume of PA, MVPA, LPA and SB with incident CKD among individuals with low and normal muscle mass.
**Table S7.** Associations of the accelerometer‐measured total volume of PA, MVPA, LPA and SB with incident CKD among individuals with mild to moderate low and normal muscle mass.
**Table S8.** Associations of the accelerometer‐measured total volume of PA, MVPA, LPA and SB with incident CKD stratified by different walk pace.
**Table S9.** Associations of the accelerometer‐measured total volume of PA with incident CKD stratified by subgroups.
**Table S10.** Sensitive analyses of associations of total volume of PA, MVPA, LPA and SB with incident CKD by using competing risk regression.
**Figure S4.** Sensitivity analyses of dose–response associations of total volume of PA, MVPA, LPA and SB with incident CKD after excluding participants with missing data on covariables.
**Table S11.** Sensitivity analyses of associations of total volume of PA, MVPA, LPA and SB with incident CKD after excluding participants with missing data on covariables.
**Figure S5.** Sensitivity analyses of dose–response associations of total volume of PA, MVPA, LPA and SB with incident CKD after excluding events occurring within the first 2 years.
**Table S12.** Sensitivity analyses of associations of total volume of PA, MVPA, LPA and SB with incident CKD after excluding events occurring within the first 2 years.

**Data S1.** Supplementary reference.

## Data Availability

Individual‐level data from the UK Biobank are not publicly available due to their policy, but the data will be made available after the application of the UK Biobank (https://www.ukbiobank.ac.uk/).
