# Supplementary material for: Device‐Measured Physical Activity, Sedentary Behaviour and Risk of Chronic Kidney Diseases Across Levels of Grip Strength
Source: J Cachexia Sarcopenia Muscle. 2025 Feb 16;16(1):e13726. doi: 10.1002/jcsm.13726 (PMC11830631; doi:10.1002/jcsm.13726)
Supplement: Supplementary file 1 — Figure S1. Flow chart of enrolment. Method S1. Formulas. Method S2. Detailed information of the muscle mass and quality. Method S3. Detailed information of the percentage of excess risk mediated (PERM). Figure S2. Frequency of total PA, MVPA, LPA and sedentary behaviour (SB). Table S1. Information about exposures, outcomes, covariates and mediators. Table S2. The number (percentage) of participants with missing covariate data in the analytic sample. Table S3. Baseline characteristics of the study participants stratified by MVPA. Table S4. Baseline characteristics of the study participants stratified by LPA. Table S5. Baseline characteristics of the study participants stratified by SB. Figure S3. Proportions attributable to different risk factors of CKD. Table S6. Associations of the accelerometer‐measured total volume of PA, MVPA, LPA and SB with incident CKD among individuals with low and normal muscle mass. Table S7. Associations of the accelerometer‐measured total volume of PA, MVPA, LPA and SB with incident CKD among individuals with mild to moderate low and normal muscle mass. Table S8. Associations of the accelerometer‐measured total volume of PA, MVPA, LPA and SB with incident CKD stratified by different walk pace. Table S9. Associations of the accelerometer‐measured total volume of PA with incident CKD stratified by subgroups. Table S10. Sensitive analyses of associations of total volume of PA, MVPA, LPA and SB with incident CKD by using competing risk regression. Figure S4. Sensitivity analyses of dose–response associations of total volume of PA, MVPA, LPA and SB with incident CKD after excluding participants with missing data on covariables. Table S11. Sensitivity analyses of associations of total volume of PA, MVPA, LPA and SB with incident CKD after excluding participants with missing data on covariables. Figure S5. Sensitivity analyses of dose–response associations of total volume of PA, MVPA, LPA and SB with incident CKD after excluding events occurrin [file JCSM-16-e13726-s001.docx]

**Supplementary Material**

**Title: Device-measured physical activity, sedentary behavior, and risk of chronic kidney diseases across levels of grip strength**

| Supplemental Figure S1 | Flow chart of enrollment | 3 |
| --- | --- | --- |
| Supplemental Methods 1 | Formulas | 4 |
| Supplemental Methods 2 | Detailed information of the muscle mass and quality | 4 |
| Supplemental Methods 3 | Detailed information of the percentage of excess risk mediated (PERM) | 5 |
| Supplemental Figure S2 | Frequency of total PA, MVPA, LPA, and sedentary behavior (SB) | 7 |
| Supplemental Table S1 | Information about exposures, outcomes, covariates, and mediators | 8 |
| Supplemental Table S2 | The number (percentage) of participants with missing covariate data in the analytic sample | 10 |
| Supplemental Table S3 | Baseline characteristics of the study participants stratified by MVPA | 11 |
| Supplemental Table S4 | Baseline characteristics of the study participants stratified by LPA | 15 |
| Supplemental Table S5 | Baseline characteristics of the study participants stratified by SB | 19 |
| Supplemental Figure S3 | Proportions attributable to different risk factors of CKD | 23 |
| Supplemental Table S6 | Associations of the accelerometer-measured total volume of PA, MVPA, LPA and SB with incident CKD among individuals with low and normal muscle mass | 25 |
| Supplemental Table S7 | Associations of the accelerometer-measured total volume of PA, MVPA, LPA and SB with incident CKD among individuals with mild to moderate low and normal muscle mass | 27 |
| Supplemental Table S8 | Associations of the accelerometer-measured total volume of PA, MVPA, LPA and SB with incident CKD stratified by different walk pace | 29 |
| Supplemental Table S9 | Associations of the accelerometer-measured total volume of PA with incident CKD stratified by subgroups | 31 |
| Supplemental Table S10 | Sensitive analyses of associations of total volume of PA, MVPA, LPA, and SB with incident CKD by using competing risk regression | 34 |
| Supplemental Figure S4 | Sensitivity analyses of dose-response associations of total volume of PA, MVPA, LPA, and SB with incident CKD after excluding participants with missing data on covariables | 36 |
| Supplemental Table S11 | Sensitivity analyses of associations of total volume of PA, MVPA, LPA, and SB with incident CKD after excluding participants with missing data on covariables | 38 |
| Supplemental Figure S5 | Sensitivity analyses of dose-response associations of total volume of PA, MVPA, LPA, and SB with incident CKD after excluding events occurring within the first two years | 40 |
| Supplemental Table S12 | Sensitivity analyses of associations of total volume of PA, MVPA, LPA, and SB with incident CKD after excluding events occurring within the first two years | 42 |


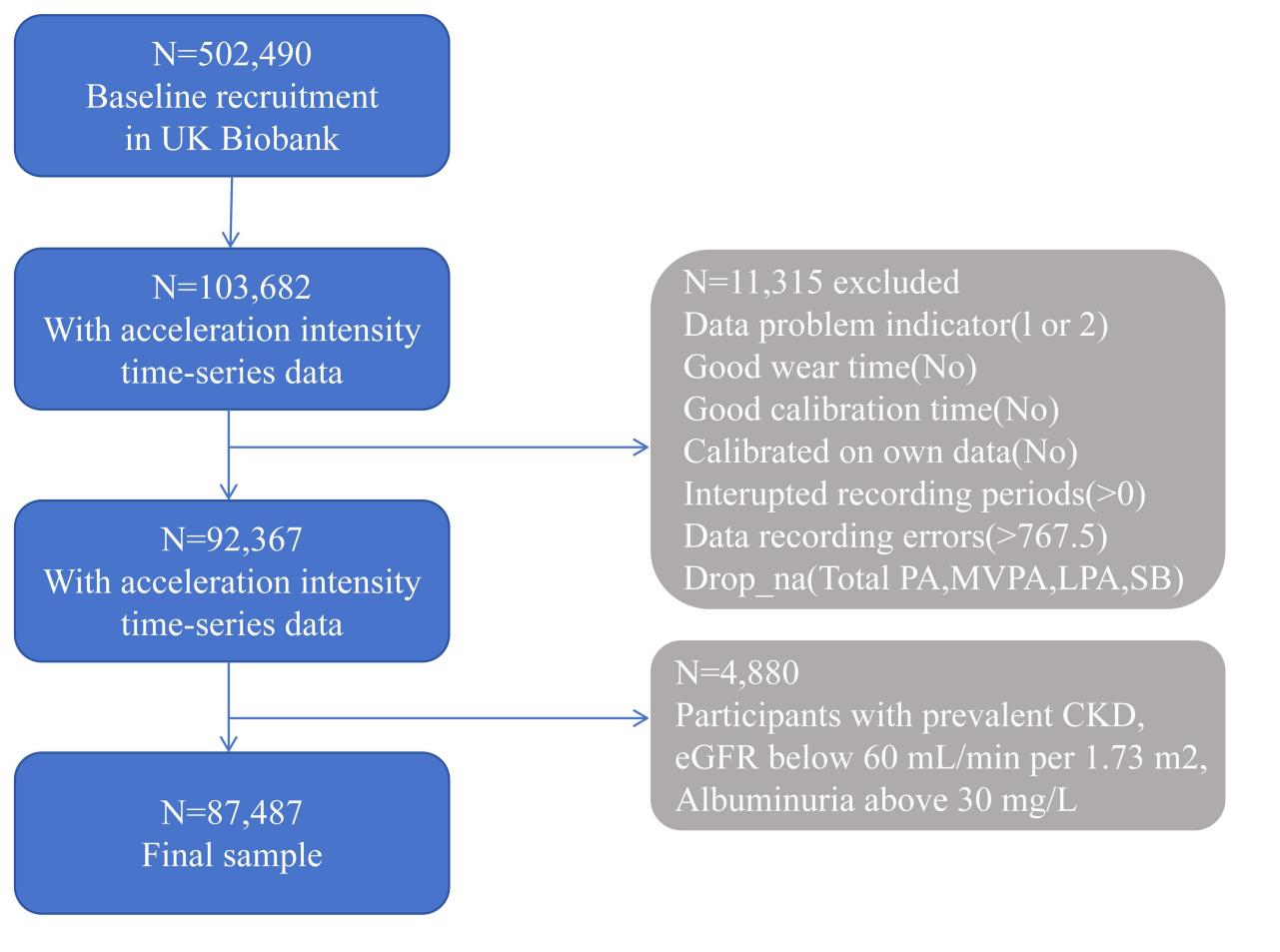


**Supplemental Figure S1. Flow chart of enrollment**

CKD, chronic kidney disease; eGFR, estimated glomerular filtration rate; LPA, light physical activity; MVPA, moderate-to-vigorous intensity physical activity; PA, physical activity; SB, sedentary behaviour.

**Supplemental Methods 1. Formulas**

1. Estimated glomerular filtration rate (eGFR) was calculated by the Chronic Kidney Disease Epidemiology Collaboration equation^1^:

For women, $eGFR=144\times{(\frac{serum creatinie}{62})}^{-0.329}\times{(0.993)}^{age}$ if serum creatinine ≤ 62 mg/dL,

$eGFR=144\times{(\frac{serum creatinie}{62})}^{-1.209}\times{(0.993)}^{age}$ if serum creatinine > 62 mg/dL;

For men, $eGFR=141\times{(\frac{serum creatinie}{80})}^{-0.411}\times{(0.993)}^{age}$ if serum creatinine ≤ 80 mg/dL,

$eGFR=141\times{(\frac{serum creatinie}{80})}^{-1.209}\times{(0.993)}^{age}$ if serum creatinine > 80 mg/dL.

2. Population-attributable fraction (PAF) was calculated using the following formula^2^:

$$PAF=\frac{p*\left( HR-1 \right)}{1+p*(HR-1)}$$

where p is the prevalence of physical inactivity in the population, and HR is the adjusted HR of CKD associated with physical inactivity.

Supplemental Methods 2 Detailed information of the muscle mass and quality

**Muscle mass：**Muscle mass was assessed using bioelectrical impedance analysis (Tanita BC 418MA Body Fat Analyser). According to previous studies,^3-5^ we calculated the appendicular lean soft tissue (ALST). The equation is as follows: ALST (kg) = (0.958 × [appendicular fat free mass (kg)]) − (0.166 × S) − 0.308, with S taking the value 0 if female and 1 if male.^5^ We defined low muscle mass as follows: ALST/height^2^ < 6.95 kg/m^2^ for men and <5.30 kg/m^2^ for women, or ALST/BMI <0.84 for men and <0.55 for women. We also used an alternative criterion^4^ to define mild to moderate low muscle mass (ALST/height2 of <7.98 kg/m2 for men and <6.15 kg/m2 for women, or low ALST/BMI of <0.94 for men and <0.64 for women).

**Quality**: The UK Biobank does not contain an objective measure of gait speed, as specified by the European Working Group on Sarcopenia in Older People (EWGSOP) and the Foundation for the National Institutes of Health Sarcopenia Project (FNIHSP) criteria. As a surrogate marker of poor gait speed and low performance, we used walk pace.^3^ The walking pace was self‐reported via a touchscreen‐based questionnaire by answering the question “How would you describe your usual walking pace? 1) Slow pace, 2) Steady/average pace, and 3) Brisk pace”.

**Supplemental Methods 3. Detailed information of the percentage of excess risk mediated (PERM)**

The percentage of excess risk mediated (PERM) was calculated to assess the extent to which baseline covariates explained the association as follows: 1) health behaviours (smoking, alcohol intake frequency, healthy diet score, and sleep duration; 2) inflammatory factors (C-reaction protein and neutrophil-to-lymphocyte ratio); 3) physiological factors (BMI, grip strength, Hb1Ac, eGFR, urate); 4) psychological factors (ever seeking help from physicians due to anxiety or depressive symptoms, history of depression, and history of anxiety, and use of antipsychotics); 5) comorbidities and medication (hypertension, high cholesterol, history of cancers, history of diabetes, history of coronary heart disease, use of cholesterol-lowering medication, and use of antihypertensive medication). For each group of mediators, we estimated the percentage of PERM as:

PERM= $\frac{\begin{aligned} \mathrm{HR} (age, sex, ethnicity, assessment center, \\ socioeconomic factors \\ adjusted)- \\ \mathrm{HR} (age, sex, ethnicity, assessment center, \\ socioeconomic factors \\ \\ and risk factors adjusted) \end{aligned}}{\begin{aligned} HR(age, sex, ethnicity, assessment center, \\ socioeconomic factors \\ adjusted)-1 \\ \end{aligned}}$ *100%

**References**:

1. Levey AS, Stevens LA, Schmid CH, Zhang YL, Castro AF 3rd, Feldman HI, Kusek JW, Eggers P, Van Lente F, Greene T, Coresh J; CKD-EPI (Chronic Kidney Disease Epidemiology Collaboration). A new equation to estimate glomerular filtration rate. Ann Intern Med. 2009 May 5;150(9):604-12.

2. Eide GE. Attributable fractions for partitioning risk and evaluating disease prevention: a practical guide. Clin Respir J. 2008 Oct;2 Suppl 1:92-103.

3. Wilkinson TJ, Miksza J, Yates T, Lightfoot CJ, Baker LA, Watson EL, Zaccardi F, Smith AC. Association of sarcopenia with mortality and end-stage renal disease in those with chronic kidney disease: a UK Biobank study. J Cachexia Sarcopenia Muscle. 2021 Jun;12(3):586-598.

4. Kiss N, Prado CM, Daly RM, Denehy L, Edbrooke L, Baguley BJ, Fraser SF, Khosravi A, Abbott G. Low muscle mass, malnutrition, sarcopenia, and associations with survival in adults with cancer in the UK Biobank cohort. J Cachexia Sarcopenia Muscle. 2023 Aug;14(4):1775-1788.

5. Dodds RM, Granic A, Robinson SM, Sayer AA. Sarcopenia, long-term conditions, and multimorbidity: findings from UK Biobank participants. J Cachexia Sarcopenia Muscle. 2020 Feb;11(1):62-68


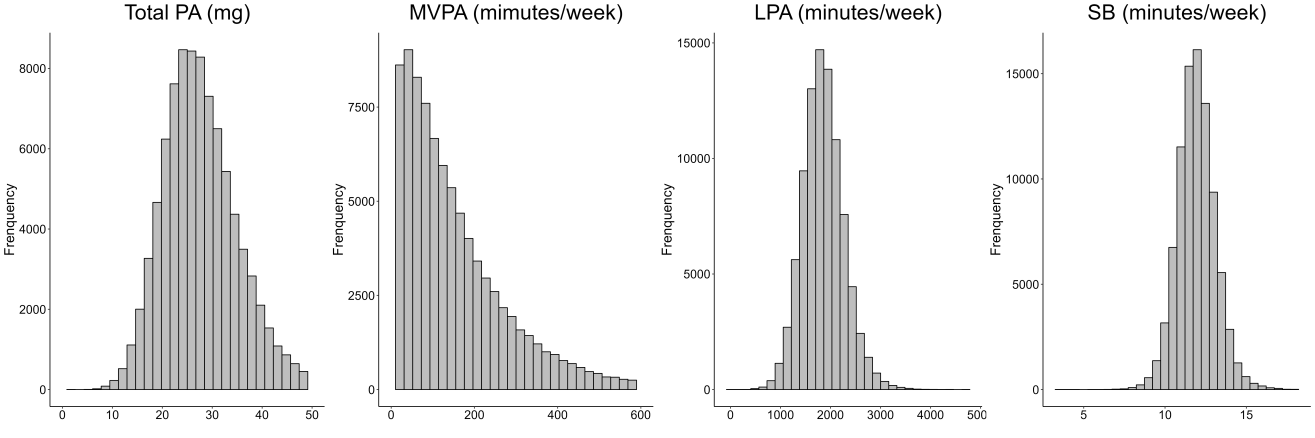
**Supplemental Figure S2. Frequency of total PA, MVPA, LPA, and SB**

PA, physical activity; MVPA, moderate-to-vigorous physical activity; LPA, light physical activity; SB, sedentary behaviour

**Supplemental Table S1**. Information about exposures, outcomes, covariates, and mediators

| Variables | Field ID | ICD-10 code |
| --- | --- | --- |
| CKD |  | N03, N06, N08, N11, N12, N13,  N14, N15, N16, N18, N19, N20, N21, E85.3, N16.5, Q60.1, T82.4, T86.1, Y60.2, Y61.2, Y62.2, Y84.1, Z49.0, Z49.1, Z49.2, Z94.0, and Z99.2. |
| Age at accelerometry | 21003 |  |
| Sex | 31 |  |
| Ethnicity | 21000 |  |
| Townsend deprivation index | 189 |  |
| Recruitment center | 54 |  |
| Education level | 6138 |  |
| Season of accelerometer wear | 90010 |  |
| Obesity status | 21001 |  |
| Healthy diet score | 1289/1299/1309/1319/1329/1339/1349/1369/1379/1389 |  |
| Sleep duration | 90001 |  |
| Grip strength | 46/47 |  |
| Smoking status | 20116 |  |
| Alcohol consumption | 1558 |  |
| HbA1c | 30750 |  |
| eGFR | 21003/31/30700 |  |
| Urate | 30880 |  |
| CRP | 30170 |  |
| NLR | 30140/30120 |  |
| Hypertension | 6150 | I10 |
| High cholesterol | 20002 | E78 |
| History of diabetes | 2443 | E10-E14 |
| History of coronary heart diseases |  | I20-25 |
| History of cancers | 2453 | C00-C97 |
| Depression |  | I60-I64; I69 |
| Anxiety |  | F32/F33/F34/F38/F39 |
| Ever seeking help from physicians due to anxiety or depressive symptoms. | 20499 |  |
| Use of blood glucose-lowering medications | 20003/6153/6177 |  |
| Use of cholesterol-lowering medications | 20003 |  |
| Use of blood pressure-lowering medications | 20003/6153/6177 |  |
| Use of psychotropic medications | 20003/6153/6177 |  |

CKD, chronic kidney disease; CRP, C-reactive protein; ICD-10, International Classification of Diseases; eGFR, estimated glomerular filtration rate; NLR, neutrophil to lymphocyte ratio

**Supplemental Table S2. The number (percentage) of participants with missing covariate data in the analytic sample**

| Covariates | N | % |
| --- | --- | --- |
| Age at accelerometry | 0 | 0.0% |
| Sex | 0 | 0.0% |
| Ethnicity | 299 | 0.3% |
| Townsend deprivation index | 96 | 0.1% |
| Recruitment center | 0 | 0.0% |
| Education level | 497 | 0.6% |
| Season of accelerometer wear | 0 | 0.0% |
| Obesity status | 146 | 0.2% |
| Healthy diet score (median) | 41 | 0.1% |
| Sleep duration (hours/day) | 0 | 0.0% |
| Grip strength (kg) | 339 | 0.4% |
| Smoking status | 201 | 0.2% |
| Alcohol consumption | 61 | 0.1% |
| HbA1c (mmol/mol) | 5,134 | 5.9% |
| eGFR (ml/min per 1.73 m^2^) | 4,637 | 5.3% |
| Urate ($\boldsymbol{\mu}$mol/l) | 4,995 | 5.7% |
| CRP | 5,250 | 6.0% |
| NLR | 3,263 | 3.7% |
| Hypertension | 100 | 0.1% |
| High cholesterol | 0 | 0.0% |
| History of diabetes | 119 | 0.1% |
| History of coronary heart diseases | 0 | 0.0% |
| History of cancers | 157 | 0.2% |
| Depression | 0 | 0.0% |
| Anxiety | 0 | 0.0% |
| Ever seeking help from physicians due to anxiety or depressive symptoms. | 25,370 | 29.0% |
| Use of blood glucose-lowering medications | 294 | 0.3% |
| Use of cholesterol-lowering medications | 294 | 0.3% |
| Use of blood pressure-lowering medications | 430 | 0.5% |
| Use of psychotropic medications | 24,286 | 27.8% |

CRP, C-reactive protein; eGFR, estimated glomerular filtration rate; NLR, neutrophil to lymphocyte ratio.

**Supplemental Table S3. Baseline characteristics of the study participants stratified by MVPA**

| **Characteristics** | **Overall (N=87,487)** | **MVPA (minutes/week)** | | **P value** |
| --- | --- | --- | --- | --- |
|  |  | **Not meeting the recommended level (N=52,739)** | **Meeting the recommended level (N=34,748)** |  |
| Age at accelerometry (years) [mean (SD)] | 62.3 (7.8) | 63.5 (7.7) | 60.4 (7.7) | < 0.001 |
| Female [n (%)] | 50062 (57.2) | 32,095 (60.9) | 17,967 (51.7) | < 0.001 |
| White ethnicity [n (%)] | 84855 (97.0) | 51,156 (97.0) | 33,699 (97.0) | 0.899 |
| Townsend deprivation index (median [IQR]) ^a^ | -2.5 [-3.8, -0.2] | -2.5 [-3.8, -0.3] | -2.4 [-3.8, -0.1] | < 0.001 |
| Recruitment center [n (%)] |  |  |  | <0.001 |
| England | 78,502 (89.7) | 47,286 (89.7) | 31,216 (89.8) |  |
| Scotland | 3,293 (3.8) | 2,188 (4.1) | 1,105 (3.2) |  |
| Wales | 5,692 (6.5) | 3,265 (6.2) | 2,427 (7.0) |  |
| Education level [n (%)] |  |  |  | < 0.001 |
| Degree or above | 38,497 (44.0) | 21,152 (40.1) | 17,345 (49.9) |  |
| Any other qualification | 41,831 (47.8) | 26,465 (50.2) | 15,366 (44.2) |  |
| No qualification | 7,159 (8.2) | 5,122 (9.7) | 2,037 (5.9) |  |
| Season of accelerometer wear [n (%)] |  |  |  | < 0.001 |
| Spring | 19,764 (22.6) | 11,467 (21.7) | 8,297 (23.9) |  |
| Summer | 22,825 (26.1) | 13,542 (25.7) | 9,283 (26.7) |  |
| Autumn | 26,171 (29.9) | 15,851 (30.1) | 10,320 (29.7) |  |
| Winter | 18,727 (21.4) | 11,879 (22.5) | 6,848 (19.7) |  |
| Obesity status [n (%)] |  |  |  | < 0.001 |
| Underweight or normal weight | 35,151 (40.2) | 17,569 (33.3) | 17,582 (50.6) |  |
| Overweight | 35,938 (41.1) | 22,396 (42.5) | 13,542 (39.0) |  |
| Obese | 16,398 (18.7) | 12,774 (24.2) | 3,624 (10.4) |  |
| Healthy diet score (median [IQR]) | 3.0 [2.0, 4.0] | 3.0 [2.0, 3.0] | 3.0 [2.0, 4.0] | < 0.001 |
| Sleep duration (hours/day) [mean (SD)] | 7.3 (0.9) | 7.3 (0.9) | 7.2 (0.9) | < 0.001 |
| Grip strength (kg) [mean (SD)] | 30.3 (10.6) | 29.3 (10.6) | 31.9 (10.4) | < 0.001 |
| Smoking status [n (%)] |  |  |  | < 0.001 |
| Never | 50,565 (57.8) | 29,554 (56.0) | 21,011 (60.5) |  |
| Previous | 31,463 (36.0) | 19,413 (36.8) | 12,050 (34.7) |  |
| Current | 5,459 (6.2) | 3,772 (7.2) | 1,687 (4.9) |  |
| Alcohol consumption [n (%)] |  |  |  | < 0.001 |
| Not current | 5158 (5.9) | 3,481 (6.6) | 1,677 (4.8) |  |
| Two or less times a week | 40414 (46.2) | 25286 (47.9) | 15,128 (43.5) |  |
| Three or more times a week | 41915 (47.9) | 23,972 (45.5) | 17,943 (51.6) |  |
| HbA1c (mmol/mol) [mean (SD)] | 35.3 (5.3) | 35.8 (5.8) | 34.6 (4.5) | < 0.001 |
| HbA1c (%) [mean (SD)] | 5.4 (0.5) | 5.4 (0.5) | 5.3 (0.4) | < 0.001 |
| eGFR (ml/min per 1.73 m^2^) [mean (SD)] | 92.0 (7.4) | 91.1 (7.4) | 93.2 (7.4) | < 0.001 |
| Urate ($\mu$mol/l) [mean (SD)] | 301.0 (77.1) | 303.9 (77.8) | 296.5 (75.8) | < 0.001 |
| CRP (mgl/L) [mean (SD)] | 2.20 (3.81) | 2.5 (4.1) | 1.7 (3.3) | < 0.001 |
| NLR (%) [mean (SD)] | 2.33 (1.15) | 2.3 (1.2) | 2.3 (1.1) | < 0.001 |
| Hypertension [n (%)] | 22,576 (25.8) | 15,940 (30.2) | 6,636 (19.1) | < 0.001 |
| High cholesterol [n (%)] | 11,822 (13.5) | 8,532 (16.2) | 3,290 (9.5) | < 0.001 |
| Depression [n (%)] | 7,714 (8.8) | 5,046 ( 9.6) | 2,668 (7.7) | < 0.001 |
| Anxiety [n (%)] | 767 (0.9) | 555 (1.1) | 212 (0.6) |  |
| Seeking help from physicians [n (%)] | 33,675 (38.5) | 20,826 (39.5) | 12,849 (37.0) | < 0.001 |
| History of diabetes [n (%)] | 3,763 (4.3) | 2,952 (5.6) | 811 (2.3) | < 0.001 |
| History of coronary heart diseases [n (%)] | 4,075 (4.7) | 3,053 (5.8) | 1,022 (2.9) | < 0.001 |
| History of cancers [n (%)] | 12,451 (14.2) | 8,251 (15.6) | 4,200 (12.1) |  |
| Medication usage [n (%)] |  |  |  | < 0.001 |
| Use of blood glucose-lowering medications | 2,056 (2.4) | 1,661 (3.1) | 395 (1.1) |  |
| Use of cholesterol-lowering medications | 14,358 (16.4) | 10,368 (19.7) | 3,990 (11.5) |  |
| Use of blood pressure-lowering medications | 15,906 (18.2) | 11,661 (22.1) | 4,245 (12.2) |  |
| Use of psychotropic medications | 2,024 (2.3) | 1,389 (2.6) | 635 (1.8) |  |

Data are presented as mean (SD), or median [IQR], or n (%).

^a^ Townsend deprivation index was calculated based on the preceding national census output areas prior to participants joining UK Biobank.

CRP, C-reactive protein; eGFR, estimated glomerular filtration rate; IQR, Interquartile range; NLR, neutrophil to lymphocyte ratio; SD, standard deviation.

**Supplemental Table S4. Baseline characteristics of the study participants stratified by LPA**

| **Characteristics** | **Overall (N=87,487)** | **LPA (minutes/week)** | | | **P value** |
| --- | --- | --- | --- | --- | --- |
|  |  | **Low (N=29,162)** | **Moderate (N=29,163)** | **High (N=29,162)** |  |
| Age at accelerometry (years) [mean (SD)] | 62.3 (7.8) | 62.5 (7.9) | 62.2 (7.8) | 62.1 (7.8) | < 0.001 |
| Female [n (%)] | 50062 (57.2) | 13,453 (46.1) | 17,173 (58.9) | 19,436 (66.6) | < 0.001 |
| White ethnicity [n (%)] | 84855 (97.0) | 28,371 (97.3) | 28,318 (97.1) | 28,166 (96.6) | < 0.001 |
| Townsend deprivation index (median [IQR]) ^a^ | -2.5 [-3.8, -0.2] | -2.3 [-3.8, 0.2] | -2.5 [-3.9, -0.4] | -2.5 [-3.9, -0.4] | < 0.001 |
| Recruitment center [n (%)] |  |  |  |  | < 0.001 |
| England | 78,502 (89.7) | 25,984 (89.1) | 26,218 (89.9) | 26,300 (90.2) |  |
| Scotland | 3,293 (3.8) | 1,077 (3.7) | 1,085 (3.7) | 1,131 (3.9) |  |
| Wales | 5,692 (6.5) | 2,101 (7.2) | 1,860 (6.4) | 1,731 (5.9) |  |
| Education level [n (%)] |  |  |  |  | < 0.001 |
| Degree or above | 38,497 (44.0) | 13,431 (46.1) | 12,933 (44.3) | 12,133 (41.6) |  |
| Any other qualification | 41,831 (47.8) | 13,159 (45.1) | 13,923 (47.7) | 14,749 (50.6) |  |
| No qualification | 7,159 (8.2) | 2,572 (8.8) | 2,307 (7.9) | 2,280 (7.8) |  |
| Season of accelerometer wear [n (%)] |  |  |  |  | < 0.001 |
| Spring | 19,764 (22.6) | 6501 (22.3) | 6,678 (22.9) | 6,585 (22.6) |  |
| Summer | 22,825 (26.1) | 6533 (22.4) | 7,548 (25.9) | 8,744 (30.0) |  |
| Autumn | 26,171 (29.9) | 8918 (30.6) | 8,860 (30.4) | 8,393 (28.8) |  |
| Winter | 18,727 (21.4) | 7210 (24.7) | 6,077 (20.8) | 5,440 (18.7) |  |
| Obesity status [n (%)] |  |  |  |  | < 0.001 |
| Underweight or normal weight | 35,151 (40.2) | 9,916 (34.0) | 11,992 (41.1) | 13,243 (45.4) |  |
| Overweight | 35,938 (41.1) | 12,442 (42.7) | 12,061 (41.4) | 11,435 (39.2) |  |
| Obese | 16,398 (18.7) | 6,804 (23.3) | 5,110 (17.5) | 4,484 (15.4) |  |
| Healthy diet score (median [IQR]) | 3.0 [2.0, 4.0] | 3.0 [2.0, 3.0] | 3.0 [2.0, 4.0] | 3.0 [2.0, 4.0] | < 0.001 |
| Sleep duration (hours/day) [mean (SD)] | 7.3 (0.9) | 7.5 (0.9) | 7.3 (0.8) | 7.0 (0.9) | < 0.001 |
| Grip strength (kg) [mean (SD)] | 30.3 (10.6) | 32.0 (10.9) | 30.1 (10.4) | 28.7 (10.2) | < 0.001 |
| Smoking status [n (%)] |  |  |  |  | < 0.001 |
| Never | 50,565 (57.8) | 16,530 (56.7) | 16,948 (58.1) | 17,087 (58.6) |  |
| Previous | 31,463 (36.0) | 10,621 (36.4) | 10,521 (36.1) | 10,321 (35.4) |  |
| Current | 5,459 (6.2) | 2,011 (6.9) | 1,694 (5.8) | 1,754 (6.0) |  |
| Alcohol consumption [n (%)] |  |  |  |  | < 0.001 |
| Not current | 5158 (5.9) | 1,738 (6.0) | 1,599 (5.5) | 1,821 (6.2) |  |
| Two or less times a week | 40414 (46.2) | 13,702 (47.0) | 13,327 (45.7) | 13,385 (45.9) |  |
| Three or more times a week | 41915 (47.9) | 13,722 (47.1) | 14,237 (48.8) | 13,956 (47.9) |  |
| HbA1c (mmol/mol) [mean (SD)] | 35.3 (5.3) | 35.4 (5.8) | 35.2 (5.2) | 35.4 (5.0) | < 0.001 |
| HbA1c (%) [mean (SD)] | 5.4 (0.5) | 5.4 (0.5) | 5.4 (0.5) | 5.4 (0.5) | < 0.001 |
| eGFR (ml/min per 1.73 m^2^) [mean (SD)] | 92.0 (7.4) | 91.3 (7.6) | 92.0 (7.4) | 92.6 (7.3) | < 0.001 |
| Urate ($\mu$mol/l) [mean (SD)] | 301.0 (77.1) | 315.3 (78.6) | 298.9 (75.9) | 288.8 (74.3) | < 0.001 |
| CRP (mgl/L) [mean (SD)] | 2.20 (3.81) | 2.4 (4.1) | 2.2 (3.7) | 2.0 (3.7) | < 0.001 |
| NLR (%) [mean (SD)] | 2.33 (1.15) | 2.4 (1.2) | 2.3 (1.1) | 2.3 (1.2) | < 0.001 |
| Hypertension [n (%)] | 22,576 (25.8) | 8,646 (29.6) | 7,252 (24.9) | 6,678 (22.9) | < 0.001 |
| High cholesterol [n (%)] | 11,822 (13.5) | 4,736 (16.2) | 3,817 (13.1) | 3,269 (11.2) | < 0.001 |
| Depression [n (%)] | 7,714 (8.8) | 2,665 (9.1) | 2,523 (8.7) | 2,526 (8.7) | 0.06 |
| Anxiety [n (%)] | 767 (0.9) | 267 (0.9) | 256 (0.9) | 244 (0.8) | 0.593 |
| Seeking help from physicians [n (%)] | 33,675 (38.5) | 11,030 (37.8) | 11,270 (38.6) | 11,375 (39.0) | 0.011 |
| History of diabetes [n (%)] | 3,763 (4.3) | 1,667 (5.7) | 1,080 (3.7) | 1,016 (3.5) | < 0.001 |
| History of coronary heart diseases [n (%)] | 4,075 (4.7) | 1,757 (6.0) | 1,276 (4.4) | 1,042 (3.6) | < 0.001 |
| History of cancers [n (%)] | 12,451 (14.2) | 4,377 (15.0) | 4,161 (14.3) | 3,913 (13.4) | < 0.001 |
| Medication usage [n (%)] |  |  |  |  | < 0.001 |
| Use of blood glucose-lowering medications | 2,056 (2.4) | 942 (3.2) | 592 (2.0) | 522 (1.8) |  |
| Use of cholesterol-lowering medications | 14,358 (16.4) | 5,917 (20.3) | 4,573 (15.7) | 3,868 (13.3) |  |
| Use of blood pressure-lowering medications | 15,906 (18.2) | 6,268 (21.5) | 5,035 (17.3) | 4,603 (15.8) |  |
| Use of psychotropic medications | 2,024 (2.3) | 809 (2.8) | 595 (2.0) | 620 (2.1) |  |

Data are presented as mean (SD), or median [IQR], or n (%).

^a^ Townsend deprivation index was calculated based on the preceding national census output areas prior to participants joining UK Biobank.

CRP, C-reactive protein; eGFR, estimated glomerular filtration rate; IQR, Interquartile range; NLR, neutrophil to lymphocyte ratio; SD, standard deviation.

**Supplemental Table S5. Baseline characteristics of the study participants stratified by SB**

| **Characteristics** | **Overall (N=87,487)** | **SB (minutes/week)** | | | **P value** |
| --- | --- | --- | --- | --- | --- |
|  |  | **Low (N=29,163)** | **Moderate (N=29,1626)** | **High (N=29,162)** |  |
| Age at accelerometry (years) [mean (SD)] | 62.3 (7.8) | 62.1 (7.7) | 62.3 (7.8) | 62.4 (8.0) | < 0.001 |
| Female [n (%)] | 50062 (57.2) | 19,911 (68.3) | 17,181 (58.9) | 12,970 (44.5) | < 0.001 |
| White ethnicity [n (%)] | 84855 (97.0) | 28,515 (97.8) | 28,376 (97.3) | 27,964 (95.9) | < 0.001 |
| Townsend deprivation index (median [IQR]) ^a^ | -2.5 [-3.8, -0.2] | -2.6 [-3.9, 0.7] | -2.5 [-3.9, -0.3] | -2.2 [-3.7, -0.4] | < 0.001 |
| Recruitment center [n (%)] |  |  |  |  | 0.613 |
| England | 78,502 (89.7) | 26,207 (89.9) | 26,103 (89.5) | 26,192 (89.8) |  |
| Scotland | 3,293 (3.8) | 1,093 (3.7) | 1,109 (3.8) | 1,091 (3.7) |  |
| Wales | 5,692 (6.5) | 1,863 (6.4) | 1,950 (6.7) | 1,879 (6.4) |  |
| Education level [n (%)] |  |  |  |  | < 0.001 |
| Degree or above | 38,497 (44.0) | 12,135 (41.6) | 12,883 (44.2) | 13,479 (46.2) |  |
| Any other qualification | 41,831 (47.8) | 14,620 (50.1) | 13,964 (47.9) | 13,247 (45.4) |  |
| No qualification | 7,159 (8.2) | 2,408 (8.3) | 2,315 (7.9) | 2,436 (8.4) |  |
| Season of accelerometer wear [n (%)] |  |  |  |  | < 0.001 |
| Spring | 19,764 (22.6) | 6,736 (23.1) | 6,640 (22.8) | 6,388 (21.9) |  |
| Summer | 22,825 (26.1) | 8,011 (27.5) | 7,488 (25.7) | 7,326 (25.1) |  |
| Autumn | 26,171 (29.9) | 8,602 (29.5) | 8,735 (30.0) | 8,834 (30.3) |  |
| Winter | 18,727 (21.4) | 5,814 (19.9) | 6,299 (21.6) | 6,614 (22.7) |  |
| Obesity status [n (%)] |  |  |  |  | < 0.001 |
| Underweight or normal weight | 35,151 (40.2) | 14,867 (51.0) | 11,742 (40.3) | 8,542 (29.3) |  |
| Overweight | 35,938 (41.1) | 10,923 (37.5) | 12,350 (42.3) | 12,665 (43.4) |  |
| Obese | 16,398 (18.7) | 3,373 (11.6) | 5,070 (17.4) | 7,955 (27.3) |  |
| Healthy diet score (median [IQR]) | 3.0 [2.0, 4.0] | 3.0 [2.0, 4.0] | 3.0 [2.0, 4.0] | 2.0 [2.0, 3.0] | < 0.001 |
| Sleep duration (hours/day) [mean (SD)] | 7.3 (0.9) | 7.7 (0.7) | 7.4 (0.7) | 6.8 (0.9) | < 0.001 |
| Grip strength (kg) [mean (SD)] | 30.3 (10.6) | 28.8 (10.1) | 30.1 (10.6) | 32.0 (10.9) | < 0.001 |
| Smoking status [n (%)] |  |  |  |  | < 0.001 |
| Never | 50,565 (57.8) | 17,668 (60.6) | 16,956 (58.1) | 15,941 (54.7) |  |
| Previous | 31,463 (36.0) | 10,066 (34.5) | 10,580 (36.3) | 10,817 (37.1) |  |
| Current | 5,459 (6.2) | 1,429 (4.9) | 1,626 (5.6) | 2,404 ( 8.2) |  |
| Alcohol consumption [n (%)] |  |  |  |  | 0.003 |
| Not current | 5158 (5.9) | 1,704 ( 5.8) | 1,623 (5.6) | 1,831 ( 6.3) |  |
| Two or less times a week | 40414 (46.2) | 13,465 (46.2) | 13,440 (46.1) | 13,509 (46.3) |  |
| Three or more times a week | 41915 (47.9) | 13,994 (48.0) | 14,099 (48.3) | 13,822 (47.4) |  |
| HbA1c (mmol/mol) [mean (SD)] | 35.3 (5.3) | 35.0 (4.6) | 35.2 (5.0) | 35.8 (6.3) | < 0.001 |
| HbA1c (%) [mean (SD)] | 5.4 (0.5) | 5.4 (0.4) | 5.4 (0.5) | 5.4 (0.6) | < 0.001 |
| eGFR (ml/min per 1.73 m^2^) [mean (SD)] | 92.0 (7.4) | 92.4 (7.2) | 92.0 (7.4) | 91.5 (7.8) | < 0.001 |
| Urate ($\mu$mol/l) [mean (SD)] | 301.0 (77.1) | 283.7 (72.7) | 299.0 (75.2) | 320.3 (78.8) | < 0.001 |
| CRP (mgl/L) [mean (SD)] | 2.20 (3.81) | 1.9 (3.6) | 2.1 (3.5) | 2.6 (4.2) | < 0.001 |
| NLR (%) [mean (SD)] | 2.33 (1.15) | 2.3 (1.1) | 2.3 (1.2) | 2.4 (1.1) | < 0.001 |
| Hypertension [n (%)] | 22,576 (25.8) | 6,101 (20.9) | 7,402 (25.4) | 9,073 (31.1) | < 0.001 |
| High cholesterol [n (%)] | 11,822 (13.5) | 3,165 (10.9) | 3,731 (12.8) | 4,926 (16.9) | < 0.001 |
| Depression [n (%)] | 7,714 (8.8) | 2,511 (8.6) | 2,518 (8.6) | 2,685 (9.2) | 0.016 |
| Anxiety [n (%)] | 767 (0.9) | 250 (0.9) | 243 (0.8) | 274 (0.9) | 0.350 |
| Seeking help from physicians [n (%)] | 33,675 (38.5) | 11,285 (38.7) | 11,262 (38.6) | 11,128 (38.2) | 0.354 |
| History of diabetes [n (%)] | 3,763 (4.3) | 791 (2.7) | 1,097 (3.8) | 1,875 (6.4) | < 0.001 |
| History of coronary heart diseases [n (%)] | 4,075 (4.7) | 990 (3.4) | 1,265 (4.3) | 1,820 (6.2) | < 0.001 |
| History of cancers [n (%)] | 12,451 (14.2) | 4,095 (14.0) | 4,202 (14.4) | 4,154 (14.2) | 0.445 |
| Medication usage [n (%)] |  |  |  |  | < 0.001 |
| Use of blood glucose-lowering medications | 2,056 (2.4) | 407 (1.4) | 570 (2.0) | 1,079 (3.7) |  |
| Use of cholesterol-lowering medications | 14,358 (16.4) | 3,651 (12.5) | 4,611 (15.8) | 6,096 (20.9) |  |
| Use of blood pressure-lowering medications | 15,906 (18.2) | 4,187 (14.4) | 5,180 (17.8) | 6,539 (22.4) |  |
| Use of psychotropic medications | 2,024 (2.3) | 658 (2.3) | 618 (2.1) | 748 (2.6) |  |

Data are presented as mean (SD), or median [IQR], or n (%).

^a^ Townsend deprivation index was calculated based on the preceding national census output areas prior to participants joining UK Biobank.

CRP, C-reactive protein; eGFR, estimated glomerular filtration rate; IQR, Interquartile range; NLR, neutrophil to lymphocyte ratio; SB, sedentary behaviour; SD, standard deviation

**Supplemental Table S6. Associations of the accelerometer-measured total volume of PA, MVPA, LPA and SB with incident CKD among individuals with low and normal muscle mass.**

| **Exposures** | **Low muscle mass** | | | **Normal muscle mass** | | |
| --- | --- | --- | --- | --- | --- | --- |
|  | **N** | **Incident rate**  **(per 1000 person-years)** | **HR (95%CI)** | **N** | **Incident rate**  **(per 1000 person-years)** | **HR (95%CI)** |
| **Total PA** |  |  |  |  |  |  |
| Low | 2,618 | 10.3 | 1 (reference) | 26,135 | 6.8 | 1 (reference) |
| Moderate | 1,629 | 6.4 | 0.83 (0.63-1.11) | 27,234 | 4.2 | 0.88 (0.80-0.96) |
| High | 1,188 | 5.4 | 1.04 (0.72-1.49) | 27,652 | 2.5 | 0.71 (0.63-0.79) |
| **MVPA** |  |  |  |  |  |  |
| Not meeting the recommended level | 3,746 | 8.9 | 1 (reference) | 48,302 | 5.5 | 1 (reference) |
| Meeting the recommended level | 1,689 | 6.2 | 1.12 (0.84-1.50) | 32,719 | 2.8 | 0.78 (0.71-0.86) |
| **LPA** |  |  |  |  |  |  |
| Low | 2,368 | 10.3 | 1 (reference) | 26,432 | 5.2 | 1 (reference) |
| Moderate | 1,664 | 7.0 | 0.81 (0.61-1.07) | 27,193 | 4.5 | 1.03 (0.94-1.13) |
| High | 1,403 | 5.6 | 0.64 (0.46-0.89) | 27,396 | 3.7 | 0.92 (0.83-1.02) |
| **SB** |  |  |  |  |  |  |
| Low | 1,262 | 5.9 | 1 (reference) | 27,564 | 3.5 | 1 (reference) |
| Moderate | 1,706 | 6.2 | 0.92 (0.64-1.33) | 27,146 | 4.3 | 1.09 (0.98-1.21) |
| High | 2,467 | 10.5 | 1.45 (1.02-2.05) | 26,311 | 5.6 | 1.24 (1.10-1.38) |

Hazard ratio was adjusted age for at the time of accelerometer wearing, sex, ethnicity, Townsend deprivation index, recruitment center, education level, the season of accelerometer wearing, obesity status, healthy diet score, sleep duration, grip strength, smoking status, alcohol consumption, HbA1c, eGFR, urate, CRP, NLR, hypertension, high cholesterol, history of diabetes, history of coronary heart diseases, history of cancers, use of blood glucose-lowering medications, use of blood pressure-lowering medications, use of cholesterol-lowering medications, depression, anxiety, use of psychotropic medications, and ever seeking help from physicians due to anxiety or depressive symptoms.

CKD, chronic kidney disease; CI, confidence interval; CRP, C-reactive protein; eGFR, estimated glomerular filtration rate; HR, hazard ratio; LPA, light physical activity; MVPA, moderate-to-vigorous physical activity; NLR, neutrophil to lymphocyte ratio; PA, physical activity; SB, sedentary behavior.

**Supplemental Table S7. Associations of the accelerometer-measured total volume of PA, MVPA, LPA and SB with incident CKD among individuals with mild to moderate low and normal muscle mass.**

| **Exposures** | **Mild to moderate low muscle mass** | | | **Normal muscle mass** | | |
| --- | --- | --- | --- | --- | --- | --- |
|  | **N** | **Incident rate**  **(per 1000 person-years)** | **HR (95%CI)** | **N** | **Incident rate**  **(per 1000 person-years)** | **HR (95%CI)** |
| **Total PA** |  |  |  |  |  |  |
| Low | 15,242 | 7.8 | 1 (reference) | 13,511 | 6.3 | 1 (reference) |
| Moderate | 13,213 | 4.6 | 0.85 (0.75-0.96) | 15,650 | 4.0 | 0.91 (0.80-1.03) |
| High | 11,625 | 3.2 | 0.80 (0.68-0.93) | 17,215 | 2.3 | 0.67 (0.58-0.79) |
| **MVPA** |  |  |  |  |  |  |
| Not meeting the recommended level | 25,391 | 6.5 | 1 (reference) | 26,657 | 5.1 | 1 (reference) |
| Meeting the recommended level | 14,689 | 3.5 | 0.84 (0.74-0.96) | 19,719 | 2.6 | 0.77 (0.68-0.88) |
| **LPA** |  |  |  |  |  |  |
| Low | 14,639 | 6.5 | 1 (reference) | 14,161 | 4.7 | 1 (reference) |
| Moderate | 13,166 | 5.4 | 1.01 (0.90-1.14) | 15,691 | 4.0 | 0.99 (0.87-1.14) |
| High | 12,275 | 4.1 | 0.84 (0.73-0.97) | 16,524 | 3.5 | 0.94 (0.81-1.08) |
| **SB** |  |  |  |  |  |  |
| Low | 12,180 | 3.9 | 1 (reference) | 16,646 | 3.4 | 1 (reference) |
| Moderate | 13,250 | 5.1 | 1.14 (0.98-1.31) | 15,602 | 3.8 | 1.03 (0.89-1.18) |
| High | 14,650 | 6.9 | 1.33 (1.14-1.55) | 14,128 | 5.0 | 1.19 (1.02-1.39) |

Hazard ratio was adjusted age for at the time of accelerometer wearing, sex, ethnicity, Townsend deprivation index, recruitment center, education level, the season of accelerometer wearing, obesity status, healthy diet score, sleep duration, grip strength, smoking status, alcohol consumption, HbA1c, eGFR, urate, CRP, NLR, hypertension, high cholesterol, history of diabetes, history of coronary heart diseases, history of cancers, use of blood glucose-lowering medications, use of blood pressure-lowering medications, use of cholesterol-lowering medications, depression, anxiety, use of psychotropic medications, and ever seeking help from physicians due to anxiety or depressive symptoms.

CKD, chronic kidney disease; CI, confidence interval; CRP, C-reactive protein; eGFR, estimated glomerular filtration rate; HR, hazard ratio; LPA, light physical activity; MVPA, moderate-to-vigorous physical activity; NLR, neutrophil to lymphocyte ratio; PA, physical activity; SB, sedentary behavior.

**Supplemental Table S8. Associations of the accelerometer-measured total volume of PA, MVPA, LPA and SB with incident CKD stratified by different walk pace.**

| **Exposures** | **Slow or steady walk pace** | | | **Brisk walk pace** | | |
| --- | --- | --- | --- | --- | --- | --- |
|  | **N** | **Incident rate**  **(per 1000 person-years)** | **HR (95%CI)** | **N** | **Incident rate**  **(per 1000 person-years)** | **HR (95%CI)** |
| **Total PA** |  |  |  |  |  |  |
| Low | 19,054 | 8.0 | 1 (reference) | 9,951 | 5.5 | 1 (reference) |
| Moderate | 15,551 | 4.9 | 0.89 (0.80-0.99) | 13,547 | 3.7 | 0.88 (0.75-1.02) |
| High | 12,103 | 3.2 | 0.76 (0.66-0.88) | 16,932 | 2.3 | 0.71 (0.60-0.85) |
| **MVPA** |  |  |  |  |  |  |
| Not meeting the recommended level | 32,587 | 6.6 | 1 (reference) | 19,912 | 4.4 | 1 (reference) |
| Meeting the recommended level | 14,121 | 3.4 | 0.80 (0.70-0.90) | 20,518 | 2.8 | 0.85 (0.75-0.97) |
| **LPA** |  |  |  |  |  |  |
| Low | 16,038 | 7.0 | 1 (reference) | 12,980 | 4.0 | 1 (reference) |
| Moderate | 15,265 | 5.7 | 1.01 (0.91-1.13) | 13,794 | 3.5 | 1.01 (0.87-1.18) |
| High | 15,405 | 4.3 | 0.84 (0.74-0.95) | 13,656 | 3.2 | 0.98 (0.83-1.15) |
| **SB** |  |  |  |  |  |  |
| Low | 14,293 | 4.3 | 1 (reference) | 14,774 | 3.0 | 1 (reference) |
| Moderate | 15,565 | 5.2 | 1.09 (0.96-1.24) | 13,505 | 3.5 | 1.03 (0.88-1.21) |
| High | 16,850 | 7.2 | 1.31 (1.15-1.50) | 12,151 | 4.4 | 1.14 (0.96-1.36) |

Hazard ratio was adjusted age for at the time of accelerometer wearing, sex, ethnicity, Townsend deprivation index, recruitment center, education level, the season of accelerometer wearing, obesity status, healthy diet score, sleep duration, grip strength, smoking status, alcohol consumption, HbA1c, eGFR, urate, CRP, NLR, hypertension, high cholesterol, history of diabetes, history of coronary heart diseases, history of cancers, use of blood glucose-lowering medications, use of blood pressure-lowering medications, use of cholesterol-lowering medications, depression, anxiety, use of psychotropic medications, and ever seeking help from physicians due to anxiety or depressive symptoms.

CKD, chronic kidney disease; CI, confidence interval; CRP, C-reactive protein; eGFR, estimated glomerular filtration rate; HR, hazard ratio; LPA, light physical activity; MVPA, moderate-to-vigorous physical activity; NLR, neutrophil to lymphocyte ratio; PA, physical activity; SB, sedentary behavior.

**
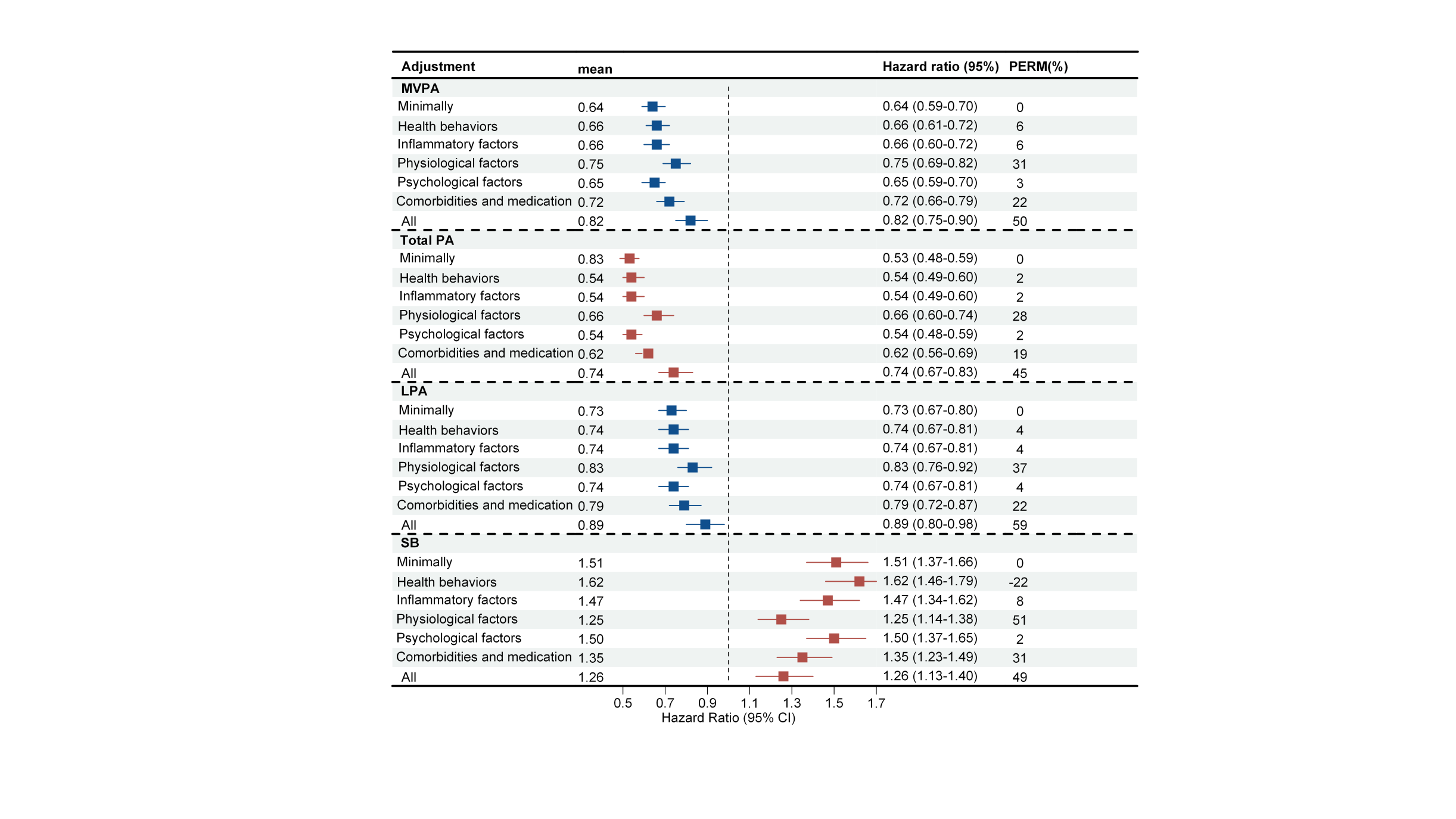
**

**Supplemental Figure S3. Proportions attributable to different risk factors of CKD**

Fully adjusted for age at the time of accelerometer wearing, sex, ethnicity, Townsend deprivation index, recruitment center, education level, the season of accelerometer wearing, obesity status, healthy diet score, sleep duration, grip strength, smoking status, alcohol consumption, HbA1c, eGFR, urate, CRP, NLR, hypertension, high cholesterol, history of diabetes, history of coronary heart diseases, history of cancers, use of blood glucose-lowering medications, use of blood pressure-lowering medications, use of cholesterol-lowering medications, depression, anxiety, use of psychotropic medications, and ever seeking help from physicians due to anxiety or depressive symptoms.

CKD, chronic kidney disease; CRP, C-reactive protein; eGFR, estimated glomerular filtration rate; LPA, light physical activity; MVPA, moderate-to-vigorous physical activity; NLR, neutrophil to lymphocyte ratio; PA, physical activity; PERM = percentage of excess risk mediated; SB, sedentary behaviour.

**Supplemental Table S9. Associations of the accelerometer-measured total volume of PA with incident CKD stratified by subgroups**

| **Total PA** | **N=87,487** | **Case/person-years** | **Incident rate**  **per 1000 person-years** | **Fully adjusted HR (95% CI)** |
| --- | --- | --- | --- | --- |
| **Age** |  |  |  |  |
| **Middle-age (<65)** |  |  |  |  |
| Low | 12,892 | 347/89,001 | 3.9 | 1.00 [Reference] |
| Moderate | 16,627 | 309/115,957 | 2.7 | 0.87 (0.74-1.02) |
| High | 20,304 | 280/142,318 | 2.0 | 0.74 (0.62-0.87) |
| **Old (≥65)** |  |  |  |  |
| Low | 16,284 | 1,064/107,476 | 9.9 | 1.00 [Reference] |
| Moderate | 12,557 | 556/84,932 | 6.6 | 0.88 (0.79-0.98) |
| High | 8,823 | 264/60,177 | 4.4 | 0.72 (0.62-0.83) |
| **Sex** |  |  |  |  |
| **Male** |  |  |  |  |
| Low | 13,795 | 783/91,972 | 8.5 | 1.00 [Reference] |
| Moderate | 11,970 | 423/81,943 | 5.2 | 0.85 (0.75-0.96) |
| High | 11,660 | 271/80,753 | 3.4 | 0.74 (0.64-0.86) |
| **Female** |  |  |  |  |
| Low | 15,381 | 628/104,506 | 6.0 | 1.00 [Reference] |
| Moderate | 17,214 | 442/118,946 | 3.7 | 0.91 (0.80-1.04) |
| High | 17,467 | 273/121,741 | 2.2 | 0.75 (0.64-0.87) |
| **Obese status** |  |  |  |  |
| **Underweight or normal weight** |  |  |  |  |
| Low | 8,294 | 269/56,403 | 4.8 | 1.00 [Reference] |
| Moderate | 11,620 | 250/80,352 | 3.1 | 0.84 (0.70-1.00) |
| High | 15,237 | 236/106,265 | 2.2 | 0.76 (0.63-0.92) |
| **Overweight** |  |  |  |  |
| Low | 12,608 | 602/85,093 | 7.1 | 1.00 [Reference] |
| Moderate | 12,523 | 402/86,080 | 4.7 | 0.88 (0.77-1.01) |
| High | 10,807 | 220/74,981 | 2.9 | 0.70 (0.59-0.82) |
| **Obese** |  |  |  |  |
| Low | 8,274 | 540/54,982 | 9.8 | 1.00 [Reference] |
| Moderate | 5,041 | 213/34,456 | 6.2 | 0.91 (0.77-1.08) |
| High | 3,083 | 88/21,248 | 4.1 | 0.81 (0.64-1.02) |
| **EGFR** |  |  |  |  |
| **< 90 mL/min per 1.73 m^2^** |  |  |  |  |
| Low | 15,015 | 1050/99,245 | 10.6 | 1.00 [Reference] |
| Moderate | 12,026 | 584/81,353 | 7.2 | 0.90 (0.81-1.00) |
| High | 8,849 | 305/60,458 | 5.0 | 0.76 (0.67-0.88) |
| **≥ 90 mL/min per 1.73 m^2^** |  |  |  |  |
| Low | 14,161 | 361/97,232 | 3.7 | 1.00 [Reference] |
| Moderate | 17,158 | 281/119,535 | 2.4 | 0.81 (0.69-0.95) |
| High | 20,278 | 239/142,036 | 1.7 | 0.68 (0.57-0.81) |

*Model 1 was adjusted for age at the time of accelerometer wearing and sex.

†Model 2 was adjusted for age at the time of accelerometer wearing, sex, ethnicity, Townsend deprivation index, recruitment center, education level, the season of accelerometer wearing, obesity status, healthy diet score, sleep duration, grip strength, smoking status, alcohol consumption, HbA1c, eGFR, urate, CRP, and NLR.

‡Model 3 was adjusted for age at the time of accelerometer wearing, sex, ethnicity, Townsend deprivation index, recruitment center, education level, the season of accelerometer wearing, obesity status, healthy diet score, sleep duration, grip strength, smoking status, alcohol consumption, HbA1c, eGFR, urate, CRP, NLR, hypertension, high cholesterol, history of diabetes, history of coronary heart diseases, history of cancers, use of blood glucose-lowering medications, use of blood pressure-lowering medications, use of cholesterol-lowering medications, depression, anxiety, use of psychotropic medications, and ever seeking help from physicians due to anxiety or depressive symptoms.

CKD, chronic kidney disease; CI, confidence interval; CRP, C-reactive protein; eGFR, estimated glomerular filtration rate; HR, hazard ratio; LPA, light physical activity; MVPA, moderate-to-vigorous physical activity; NLR, neutrophil to lymphocyte ratio; PA, physical activity

**Supplemental Table S10. Sensitive analyses of associations of total volume of PA, MVPA, LPA and SB with incident CKD by using competing risk regression**

| **Exposures** | **N** | **Case/person-years** | **Incident rate**  **per 1000 person-years** | **Model 1^*^** | **Model 2^†^** | **Model 3^‡^** |
| --- | --- | --- | --- | --- | --- | --- |
|  |  |  |  | **HR (95% CI)** | **HR (95% CI)** | **HR (95% CI)** |
| **Total PA** |  |  |  |  |  |  |
| Low | 29,176 | 1,411/196,477 | 7.2 | 1 (Reference) | 1 (Reference) | 1 (Reference) |
| Moderate | 29,184 | 865/200,889 | 4.3 | 0.71 (0.65-0.77) | 0.83 (0.76-0.91) | 0.88 (0.80-0.96) |
| High | 29,127 | 544/2024,94 | 2.7 | 0.53 (0.48-0.58) | 0.68 (0.61-0.76) | 0.74 (0.67-0.83) |
| **MVPA** |  |  |  |  |  |  |
| Not meeting the recommended level | 52,739 | 2,088/358,892 | 5.8 | 1 (Reference) | 1 (Reference) | 1 (Reference) |
| Meeting the recommended level | 34,748 | 732/240,969 | 3.0 | 0.63 (0.58-0.68) | 0.78 (0.71-0.85) | 0.82 (0.75-0.90) |
| **LPA** |  |  |  |  |  |  |
| Low | 29,162 | 1,126/198,248 | 5.7 | 1 (Reference) | 1 (Reference) | 1 (Reference) |
| Moderate | 29,163 | 933/200501 | 4.7 | 0.87 (0.80-0.95) | 0.97 (0.89-1.06) | 1.00 (0.91-1.09) |
| High | 29,162 | 761/201,111 | 3.8 | 0.73 (0.67-0.80) | 0.85 (0.77-0.94) | 0.89 (0.80-0.98) |
| **SB** |  |  |  |  |  |  |
| Low | 29,163 | 737/201,364 | 3.7 | 1 (Reference) | 1 (Reference) | 1 (Reference) |
| Moderate | 29,162 | 882/200,384 | 4.4 | 1.15 (1.05-1.27) | 1.09 (0.99-1.21) | 1.07 (0.97-1.18) |
| High | 29,162 | 1,201/198,112 | 6.1 | 1.53 (1.38-1.67) | 1.33 (1.19-1.47) | 1.26 (1.13-1.40) |

*Model 1 was adjusted for age at the time of accelerometer wearing and sex.

†Model 2 was adjusted for age at the time of accelerometer wearing, sex, ethnicity, Townsend deprivation index, recruitment center, education level, the season of accelerometer wearing, obesity status, healthy diet score, sleep duration, grip strength, smoking status, alcohol consumption, HbA1c, eGFR, urate, CRP, and NLR.

‡Model 3 was adjusted for age at the time of accelerometer wearing, sex, ethnicity, Townsend deprivation index, recruitment center, education level, the season of accelerometer wearing, obesity status, healthy diet score, sleep duration, grip strength, smoking status, alcohol consumption, HbA1c, eGFR, urate, CRP, NLR, hypertension, high cholesterol, history of diabetes, history of coronary heart diseases, history of cancers, use of blood glucose-lowering medications, use of blood pressure-lowering medications, use of cholesterol-lowering medications, depression, anxiety, use of psychotropic medications, and ever seeking help from physicians due to anxiety or depressive symptoms.

CKD, chronic kidney disease; CI, confidence interval; CRP, C-reactive protein; eGFR, estimated glomerular filtration rate; HR, hazard ratio; LPA, light physical activity; MVPA, moderate-to-vigorous physical activity; NLR, neutrophil to lymphocyte ratio; PA, physical activity; SB, sedentary behaviour;..


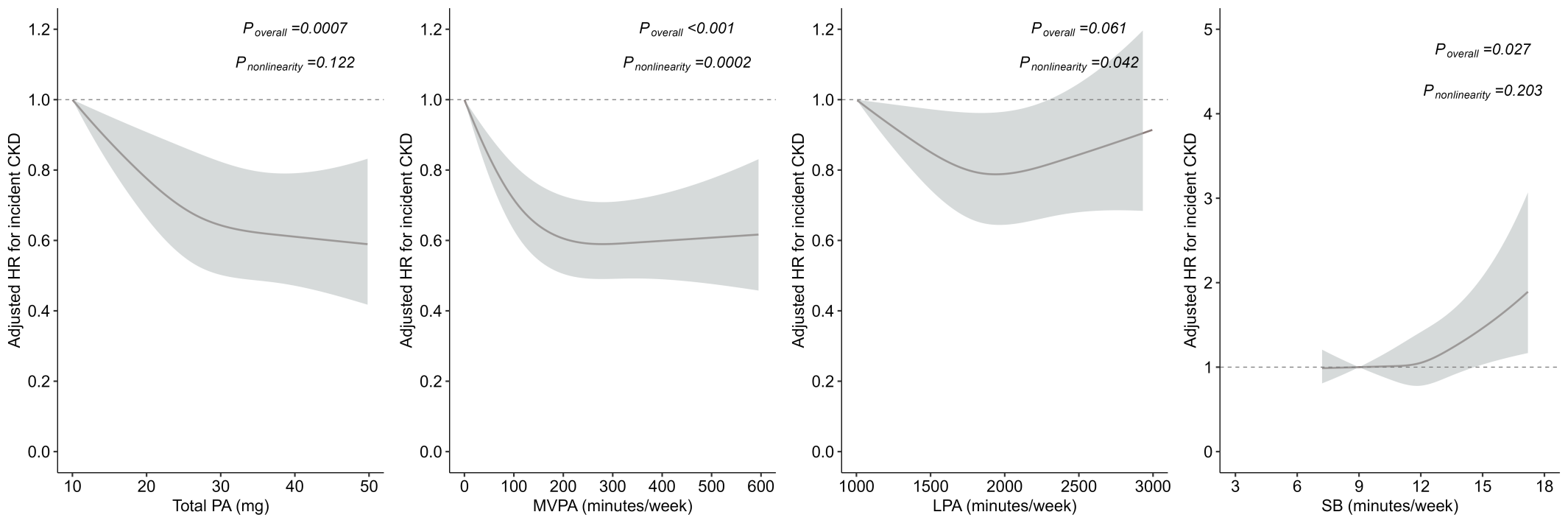


**Supplemental Figure S4.** **Sensitivity analyses of dose-response associations of total volume of PA, MVPA, LPA, and SB with incident CKD after excluding participants with missing data on covariables**

The solid line referred to the HRs from restricted cubic spline regression. Restricted cubic splines were constructed with 3 knots. Adjusted HRs (95% CI) were calculated using Cox proportional hazards regression analysis adjusted for age at the time of accelerometer wearing, sex, ethnicity, Townsend deprivation index, recruitment center, education level, the season of accelerometer wearing, obesity status, healthy diet score, sleep duration, grip strength, smoking status, alcohol consumption, HbA1c, eGFR, urate, CRP, NLR, hypertension, high cholesterol, history of diabetes, history of coronary heart diseases, history of cancers, use of blood glucose-lowering medications, use of blood pressure-lowering medications, use of cholesterol-lowering medications, depression, anxiety, use of psychotropic medications, and ever seeking help from physicians due to anxiety or depressive symptoms.

CKD, chronic kidney disease; CI, confidence interval; CRP, C-reactive protein; eGFR, estimated glomerular filtration rate; HR, hazard ratio; LPA, light physical activity; MVPA, moderate-to-vigorous physical activity; NLR, neutrophil to lymphocyte ratio; PA, physical activity; SB, sedentary behaviour.

**Supplemental Table S11. Sensitivity analyses of associations of total volume of PA, MVPA, LPA, and SB with incident CKD after excluding participants with missing data on covariables**

| **Exposures** | **N** | **Case/person-years** | **Incident rate**  **per 1000 person-years** | **Model 1^*^** | **Model 2^†^** | **Model 3^‡^** |
| --- | --- | --- | --- | --- | --- | --- |
|  |  |  |  | **HR (95% CI)** | **HR (95% CI)** | **HR (95% CI)** |
| **Total PA** |  |  |  |  |  |  |
| Low | 13,518 | 663/91,595 | 7.2 | 1 (Reference) | 1 (Reference) | 1 (Reference) |
| Moderate | 12,920 | 414/89,016 | 4.7 | 0.75 (0.66-0.85) | 0.89 (0.78-1.01) | 0.92 (0.81-1.05) |
| High | 11,927 | 248/82,776 | 3.0 | 0.57 (0.49-0.66) | 0.76 (0.65-0.89) | 0.81 (0.69-0.95) |
| **MVPA** |  |  |  |  |  |  |
| Not meeting the recommended level | 23,938 | 1005/163,383 | 6.2 | 1 (Reference) | 1 (Reference) | 1 (Reference) |
| Meeting the recommended level | 14,427 | 320/100,003 | 3.2 | 0.61 (0.53-0.69) | 0.76 (0.66-0.86) | 0.79 (0.69-0.90) |
| **LPA** |  |  |  |  |  |  |
| Low | 13,031 | 534/88,903 | 6.0 | 1 (Reference) | 1 (Reference) | 1 (Reference) |
| Moderate | 13,047 | 427/89,875 | 4.8 | 0.84 (0.74-0.95) | 0.94 (0.82-1.07) | 0.96 (0.84-1.09) |
| High | 12,287 | 364/84,609 | 4.3 | 0.78 (0.68-0.90) | 0.95 (0.82-1.10) | 0.98 (0.85-1.14) |
| **SB** |  |  |  |  |  |  |
| Low | 12,789 | 358/88,187 | 4.1 | 1 (Reference) | 1 (Reference) | 1 (Reference) |
| Moderate | 12,788 | 403/87,944 | 4.6 | 1.08 (0.94-1.25) | 0.99 (0.86-1.15) | 0.98 (0.85-1.13) |
| High | 12,788 | 564/87,256 | 6.5 | 1.45 (1.27-1.66) | 1.19 (1.02-1.39) | 1.15 (0.98-1.34) |

*Model 1 was adjusted for age at the time of accelerometer wearing and sex.

†Model 2 was adjusted for age at the time of accelerometer wearing, sex, ethnicity, Townsend deprivation index, recruitment center, education level, the season of accelerometer wearing, obesity status, healthy diet score, sleep duration, grip strength, smoking status, alcohol consumption, HbA1c, eGFR, urate, CRP, and NLR.

‡Model 3 was adjusted for age at the time of accelerometer wearing, sex, ethnicity, Townsend deprivation index, recruitment center, education level, the season of accelerometer wearing, obesity status, healthy diet score, sleep duration, grip strength, smoking status, alcohol consumption, HbA1c, eGFR, urate, CRP, NLR, hypertension, high cholesterol, history of diabetes, history of coronary heart diseases, history of cancers, use of blood glucose-lowering medications, use of blood pressure-lowering medications, use of cholesterol-lowering medications, depression, anxiety, use of psychotropic medications, and ever seeking help from physicians due to anxiety or depressive symptoms.

CKD, chronic kidney disease; CI, confidence interval; CRP, C-reactive protein; eGFR, estimated glomerular filtration rate; HR, hazard ratio; LPA, light physical activity; MVPA, moderate-to-vigorous physical activity; NLR, neutrophil to lymphocyte ratio; PA, physical activity; SB, sedentary behaviour...

**
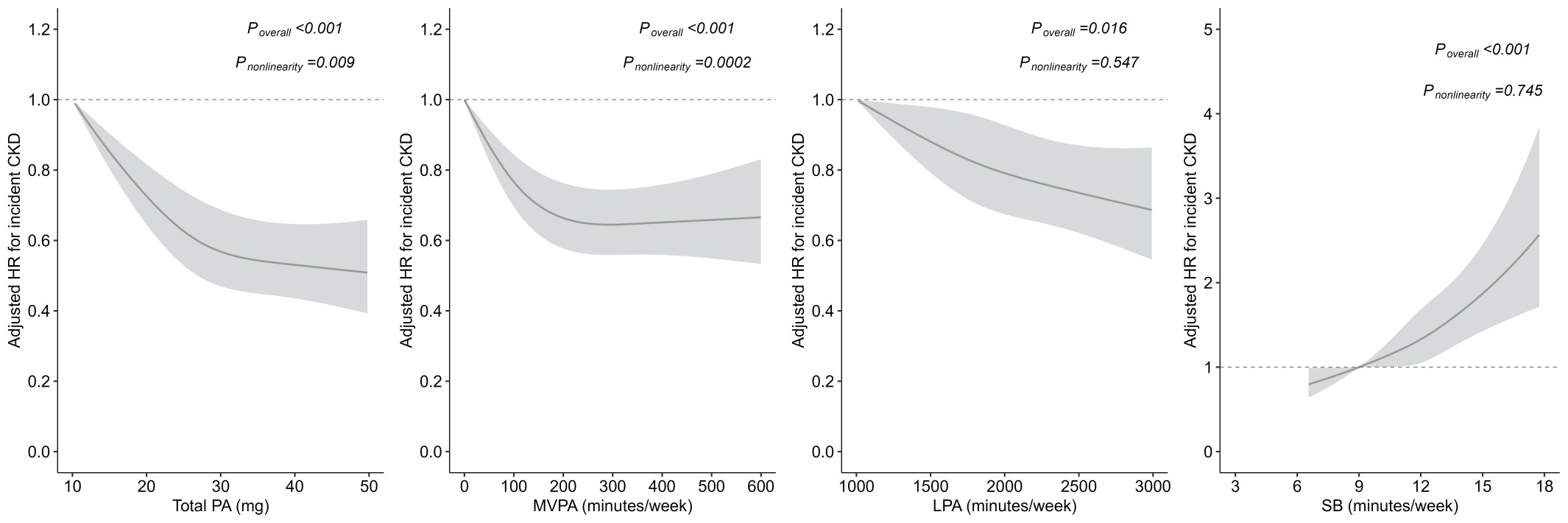
**

**Supplemental Figure S5. Sensitivity analyses of dose-response associations of total volume of PA, MVPA, LPA, and SB with incident CKD after excluding events occurring within the first two years**

The solid line referred to the HRs from restricted cubic spline regression. Restricted cubic splines were constructed with 3 knots. Adjusted HRs (95% CI) were calculated using Cox proportional hazards regression analysis adjusted for age at the time of accelerometer wearing, sex, ethnicity, Townsend deprivation index, recruitment center, education level, the season of accelerometer wearing, obesity status, healthy diet score, sleep duration, grip strength, smoking status, alcohol consumption, HbA1c, eGFR, urate, CRP, NLR, hypertension, high cholesterol, history of diabetes, history of coronary heart diseases, history of cancers, use of blood glucose-lowering medications, use of blood pressure-lowering medications, use of cholesterol-lowering medications, depression, anxiety, use of psychotropic medications, and ever seeking help from physicians due to anxiety or depressive symptoms.

CKD, chronic kidney disease; CI, confidence interval; CRP, C-reactive protein; eGFR, estimated glomerular filtration rate; HR, hazard ratio; LPA, light physical activity; MVPA, moderate-to-vigorous physical activity; NLR, neutrophil to lymphocyte ratio; PA, physical activity; SB, sedentary behaviour.

**Supplemental Table S12. Sensitivity analyses of associations of total volume of PA, MVPA, LPA and SB with incident CKD after excluding events occurring within the first two years**

| **Exposures** | **N** | **Case/person-years** | **Incident rate**  **per 1000 person-years** | **Model 1^*^** | **Model 2^†^** | **Model 3^‡^** |
| --- | --- | --- | --- | --- | --- | --- |
|  |  |  |  | **HR (95% CI)** | **HR (95% CI)** | **HR (95% CI)** |
| **Total PA** |  |  |  |  |  |  |
| Low | 28,631 | 1,110/195,895 | 5.7 | 1 (Reference) | 1 (Reference) | 1 (Reference) |
| Moderate | 28,918 | 674/200,606 | 3.4 | 0.69(0.63-0.77) | 0.81(0.74-0.90) | 0.85(0.77-0.94) |
| High | 28,950 | 433/202,285 | 2.1 | 0.53(0.47-0.59) | 0.68(0.60-0.76) | 0.73(0.65-0.83) |
| **MVPA** |  |  |  |  |  |  |
| Not meeting the recommended level | 51,978 | 1,628/358,069 | 4.6 | 1 (Reference) | 1 (Reference) | 1 (Reference) |
| Meeting the recommended level | 34,521 | 589/240,717 | 2.5 | 0.65(0.59-0.71) | 0.80(0.72-0.88) | 0.84(0.76-0.93) |
| **LPA** |  |  |  |  |  |  |
| Low | 28,711 | 871/197,782 | 4.4 | 1 (Reference) | 1 (Reference) | 1 (Reference) |
| Moderate | 28,882 | 752/200,193 | 3.8 | 0.90(0.82-1.00) | 0.99(0.90-1.10) | 1.02(0.93-1.13) |
| High | 28,906 | 594/200,811 | 3.0 | 0.73(0.66-0.81) | 0.84(0.75-0.94) | 0.87(0.78-0.98) |
| **SB** |  |  |  |  |  |  |
| Low | 28,833 | 572/200,441 | 2.9 | 1 (Reference) | 1 (Reference) | 1 (Reference) |
| Moderate | 28,833 | 701/199,735 | 3.5 | 1.18(1.06-1.32) | 1.11(0.99-1.24) | 1.09(0.97-1.22) |
| High | 28,833 | 944/198,610 | 4.8 | 1.54(1.39-1.72) | 1.31(1.17-1.48) | 1.25(1.11-1.41) |

*Model 1 was adjusted for age at the time of accelerometer wearing and sex.

†Model 2 was adjusted for age at the time of accelerometer wearing, sex, ethnicity, Townsend deprivation index, recruitment center, education level, the season of accelerometer wearing, obesity status, healthy diet score, sleep duration, grip strength, smoking status, alcohol consumption, HbA1c, eGFR, urate, CRP, and NLR.

‡Model 3 was adjusted for age at the time of accelerometer wearing, sex, ethnicity, Townsend deprivation index, recruitment center, education level, the season of accelerometer wearing, obesity status, healthy diet score, sleep duration, grip strength, smoking status, alcohol consumption, HbA1c, eGFR, urate, CRP, NLR, hypertension, high cholesterol, history of diabetes, history of coronary heart diseases, history of cancers, use of blood glucose-lowering medications, use of blood pressure-lowering medications, use of cholesterol-lowering medications, depression, anxiety, use of psychotropic medications, and ever seeking help from physicians due to anxiety or depressive symptoms.

CKD, chronic kidney disease; CI, confidence interval; CRP, C-reactive protein; eGFR, estimated glomerular filtration rate; HR, hazard ratio; LPA, light physical activity; MVPA, moderate-to-vigorous physical activity; NLR, neutrophil to lymphocyte ratio; PA, physical activity; SB, sedentary behaviour.
