# Supplementary material for: Device‐Measured Physical Activity, Sedentary Behaviour and Risk of Chronic Kidney Diseases Across Levels of Grip Strength
Source: J Cachexia Sarcopenia Muscle. 2025 Feb 16;16(1):e13726. doi: 10.1002/jcsm.13726 (PMC11830631; doi:10.1002/jcsm.13726)
Supplement: Supplementary file 2 — Data S1. Supplementary reference. [file JCSM-16-e13726-s002.docx]

**Device-measured physical activity, sedentary behavior, and risk of chronic kidney diseases across levels of grip strength**

**Supplemental references:**

S1. Reisin E, Jack AV. Obesity and hypertension: mechanisms, cardio-renal consequences, and therapeutic approaches. Med Clin North Am. 2009;93:733-51.

S2. Edwards JJ, Deenmamode AHP, Griffiths M, Arnold O, Cooper NJ, Wiles JD, et al. Exercise training and resting blood pressure: a large-scale pairwise and network meta-analysis of randomised controlled trials. Br J Sports Med. 2023;57:1317-26.

S3. Sylow L, Kleinert M, Richter EA, Jensen TE. Exercise-stimulated glucose uptake - regulation and implications for glycaemic control. Nat Rev Endocrinol. 2017;13:133-48.

S4. Chen JH, Wen CP, Wu SB, Lan JL, Tsai MK, Tai YP, et al. Attenuating the mortality risk of high serum uric acid: the role of physical activity underused. Ann Rheum Dis. 2015;74:2034-42.

S5. Cho AR, Moon JY, Kim S, An KY, Oh M, Jeon JY, et al. Effects of alternate day fasting and exercise on cholesterol metabolism in overweight or obese adults: A pilot randomized controlled trial. Metabolism. 2019;93:52-60.

S6. Del Pozo Cruz B, Ahmadi M, Inan-Eroglu E, Huang BH, Stamatakis E. Prospective Associations of Accelerometer-Assessed Physical Activity With Mortality and Incidence of Cardiovascular Disease Among Adults With Hypertension: The UK Biobank Study. J Am Heart Assoc. 2022;11:e023290.
